# Efficacy of Palm Oil Application in Tiger Puffer Diets: Growth, Body Composition, Muscle Texture, and Lipid Metabolism

**DOI:** 10.1155/2024/2709579

**Published:** 2024-08-12

**Authors:** Yuhan Fan, Haiyan Xiong, Jiahao Liu, Guoxu Liu, Qiang Ma, Yuliang Wei, Mengqing Liang, Houguo Xu

**Affiliations:** ^1^ College of Fisheries and Life Sciences Shanghai Ocean University, 999 Huchenghuan Road, Shanghai 201306, China; ^2^ State Key Laboratory of Mariculture Biobreeding and Sustainable Goods Yellow Sea Fisheries Research Institute Chinese Academy of Fishery Sciences, 106 Nanjing Road, Qingdao 266071, China

## Abstract

Palm oil, with its higher production, lower prices, and higher levels of palmitic acid and oleic acid, may have great potential for use in the aquafeed industry. In this study, with an 8-week feeding experiment, the efficacy of palm oil as a substitute for fish oil in tiger puffer feeds was comprehensively evaluated. The control diets (FO group) contained 8% marine fish oil as the main lipid source, while in the treatment diets, the added marine fish oil was replaced with palm oil at 25%, 50%, 75%, and 100%, respectively, which was named 25PO, 50PO, 75PO, and 100PO, respectively. Juvenile tiger puffers with an initial weight of 15.0 ± 0.04 g were used, with three replicate tanks of 30 juvenile fish tiger puffer for each dietary group. The fish oil replacement by palm oil did not have an adverse effect on fish growth and feeding, but the weight gain decreased by 17.3% in group PO100. Palm oil had no significant effects on fish proximate composition and muscle texture. The effects of dietary palm oil on muscle fatty acid composition were not significant, with DHA and EPA significantly lowered only in the 100PO group. In contrast, the changes in liver and intestinal fatty acid compositions in response to diets were more significant than those in the muscle. In the intestine, the replacement of more than 50% fish oil by palm oil significantly downregulated the gene expression associated with peroxisomal fatty acid *β*-oxidation and triglyceride hydrolysis, while upregulated the expression of cholesterol biosynthetic genes. In the liver, the replacement of more than 75% fish oil also significantly upregulated the cholesterol synthesis. In conclusion, palm oil can replace 75% of added marine fish oil in tiger puffer diets and does not adversely affect the growth performance, feed utilization, muscle composition, and muscle texture.

## 1. Introduction

Fish oil (FO), rich in n-3 long chain-polyunsaturated fatty acids (LC-PUFA), is a high-quality lipid source for aquafeeds [1, 2, 3]. In recent years, with the rapid expansion of aquaculture, as a limited fishery resource, FO has been in short supply and its price has soared [4, 5]. Moreover, it has been shown that a portion of the FO currently used in aquafeeds is wasted, and that FO in feeds can be partly replaced by other easily available oil sources if their fatty acid compositions can help to satisfy the requirements of aquaculture fish species [2, 3, 6, 7, 8]. The FO replacement with other land-based oils is being more and more commercially viable and has gradually become an important research topic in aquanutrition [4].

Land-based oils commonly used in feeds include two main categories, namely, animal and vegetable oils [5, 7, 9, 10, 11]. Most vegetable lipid sources have less n-3 LC-PUFA, but most of them are rich in saturated fatty acids (SFA), monounsaturated fatty acids (MUFA), and n-6 LC-PUFA. It has been shown that the richness of SFA and MUFA in the feed will limit the energy used for lipid synthesis and spare the *β*-oxidation of n-3 LC-PUFA, and thus promote the accumulation of n-3 LC-PUFA in fish tissues. This is known as the “n-3 LC-PUFA sparing effects” [12, 13, 14]. Therefore, vegetable lipid sources with extra amounts of SFA and MUFA may have greater potential for use in the aquafeed industry.

Soybean oil was the most commonly used vegetable lipid source in aquafeeds. However, the linoleic acid content of soybean oil was so high that its use in large quantities in diets caused severe linoleic acid deposition in the muscle of commercial fish, which was usually difficult to recover by refeeding with FO-based diets before harvest [15, 16]. Currently, although there is still a relatively large controversy about the role of linoleic acid in human foods, most of the studies concluded that excess linoleic acid intake can cause damage to human health [17, 18]. Compared with soybean oil, palm oil is much cheaper and has only about one-fifth of the linoleic acid level of soybean oil [19]. Therefore, palm oil could be a more suitable lipid source for FO replacement than soybean oil.

Palm oil (PO) has the largest production of vegetable oils in the world [20]. Palm oil contains high levels of SFA and MUFA, especially palmitic acid (16 : 0) and oleic acid (18 : 1n-9) [7, 9, 10, 16]. Moreover, it has been shown that the complete replacement of FO by palm oil in diets did not negatively affect the growth of a certain number of fish species, such as Atlantic salmon (*Salmo salar*) [1], rainbow trout (*Oncorhynchus mykiss*) [21], spotted seabass (*Lateolabrax japonicus*) [22], Arctic charr (*Salvelinus alpinus*) [23], and greater amberjack (*Seriola dumerili*) [24]. In Asian catfish (*Pangasius nasutus*) [25], snakehead (*Channa striatus*) [26], and Asian seabass (*L. japonicus*) [27], it was found that the fish growth performance fed a diet with PO was even better compared to those fed a diet with FO only. Nevertheless, adverse effects of PO were also found. For Indian mrigal (*Cirrhinus mrigala*), replacement of more than 25% of dietary FO by PO significantly reduced the growth [28]. For Japanese flounder (*Paralichthys olivaceus*), replacement of more than 40% of FO in diets by PO significantly inhibited the growth and feed efficiency [29]. For Chu's Croaker (*Nibea coibor*), a FO replacement level exceeding 80% by PO significantly reduced the weight gain and specific growth rate [30], and complete FO replacement by palm oil inhibited the growth performance of large yellow croaker (*Larimichthys crocea*) [31]. Therefore, the efficacy of PO in diets could be fish species-specific.

Tiger puffer (*Takifugu rubripes*) is an important and traditional farming species in East Asia [32, 33, 34, 35]. Previous studies have reported that partial or complete replacement of FO in tiger puffer diets with animal lipid sources such as poultry oil, beef tallow, lard, and Basa fish offal oil did not negatively influence the growth [36, 37, 38, 39]. FO replacement by a mixture of poultry oil and coconut oil (1 : 1) also did not adversely impact the tiger puffer growth [40]. However, complete replacement of FO with soybean oil negatively affected both the growth and feed utilization [32]. No information has been available about the efficacy of FO replacement by PO in diets of tiger puffer. Therefore, the aim of this study was to comprehensively evaluate the feasibility of PO as a substitute for FO in tiger puffer diets via a serial of growth, physiological, and quality parameters. This study may enhance the understanding of palm oil application in fish feeds.

## 2. Materials and Methods

### 2.1. Diets Preparation

Five experimental isonitrogenous (containing approximately 45% crude protein) and isolipidic (containing approximately 11% crude lipid) diets were formulated. The diets differed only in the levels of FO and PO added (Table 1). The control (FO group) diets had 8% marine FO added as the main lipid source, while in the other experimental group diets, the added marine FO was replaced with palm oil at 25%, 50%, 75%, and 100%, named group 25PO, 50PO, 75PO, and 100PO, respectively. The commercial palm oil used in this study was refined from crude palm oil, with a melting point of about 24°C (imported from Malaysia by Shijiazhuang Shenze County Oil Workshop, Shijiazhuang, China). The diets were prepared following the common processes in our lab [41]. The ingredients were first grinded and mixed, and then pelleted into pellets with diameters of 2 and 4 mm. The experimental pellets were then dried and stored at −20°C until use. The fatty acid composition of the five diets, FO, and palm oil are shown in Table 2 and Figure S1.

PO has high levels of palmitic acids (PA, 16 : 0) and oleic acids (OA, 18 : 1n-9), but has no docosahexaenoic acid (DHA, 22 : 6n-3) and eicosapentaenoic acid (EPA, 20 : 5n-3) (Figure S1). Therefore, as the level of FO replacement increased, the diets contained increased levels of PA and OA, but decreased levels of DHA and EPA (Table 2 and Figure S1). Compared to the FO group, in the 100PO group, the PA content was increased by about 58.3% and the OA content was increased by about 120%; but the DHA content was decreased by 63.0% and the EPA content was decreased by 55.3% (Table 2 and Figure S2).

### 2.2. Fish Management

The feeding experiments were conducted at Haiyang Huanghai Aquaculture Co., Ltd. (Yantai, China). A group of juvenile tiger puffer with an average initial weight of 15.0 ± 0.04 g was selected and reared in a flow-through system (seawater). The experimental fish were first fed commercial diets for 2 weeks, and after acclimation was completed, the fish were fasted for 24 hr. A total of 450 juvenile tiger puffers were weighed and randomly stocked into 15 tanks (polyethylene; appr. 200 L) with three replicate tanks for each group. Fish were fed to apparent satiation two times a day at 7 : 30 a.m. and 18 : 30 p.m. The feces and residual feeds were cleaned every day by a siphoning method. During the experiment period, in the first 2 weeks, fish were fed with experimental diets with a diameter of 2 mm, and in the latter 6 weeks, fish were fed with experimental diets with a diameter of 4 mm. The water temperature was controlled to be 23–25°C, salinity to be 28–30, dissolved oxygen to be >8 mg/L, and pH to be 7.6–7.8.

### 2.3. Collection of Samples

At the end of this experiment, the number and weight of fish in each tank were recorded at 12 hr after the last feeding. After anesthetization with eugenol (eugenol: water = 1/10,000), seven fish were selected in a random order from each tank for sampling. The blood was taken from the caudal vein to a syringe (1 mL). After being put in the refrigerator at 4°C for about 2–3 hr, the serum samples were obtained by centrifugation (4,000 r/min, 4°C, 10 min). The fish were then dissected to collect the muscle, liver, and intestinal samples. For the purpose of molecular analysis, the midintestine section was carefully separated from each of the three experimental fish in each tank. These midintestine samples were processed individually for the following molecular studies. The samples collected were immediately kept in liquid nitrogen, and then finally transferred to a −80°C storage. At the end of sampling, four whole fish were selected from each tank in a random order and put into the −20°C refrigerator, for the measurement of somatic indices and proximate composition. All handling and sampling practices in this study were reviewed and approved by the Animal Care and Use Committee of the Yellow Sea Fisheries Research Institute.

### 2.4. Proximate and Fatty Acid Composition

The analysis of proximate composition followed the methods of Association of Official Analytical Chemists (AOAC 2005): The moisture content was measured by drying at 105°C for 24 hr till constant weight; the crude protein content was measured using a Kjeldahl method (Foss 2300, Hoganas, Denmark); the crude lipid content was estimated with extraction by petroleum ether using a Soxhlet extractor (Foss Tecator, Hoganas, Sweden); the ash content was quantified by incarnating the samples in a furnace at a high temperature (550°C) for 8 hr. The total lipids in tissues were extracted using the dichloromethane:methanol (2 : 1) method.

The fatty acid composition of all tissues was measured using a gas chromatograph (GC-2010 pro, Shimadzu, Japan). The samples of muscle and intestine were lyophilized in a freeze dryer for 24 hr. The samples of the liver were handled directly with wet tissue, and the lipids were extracted with the chloroform–methanol solution. For the intestinal samples, the whole intestine after sampling of a small piece (1 cm) of midintestine from each fish was used for the fatty acid analysis, and nine intestines from each group (three per tank) were pooled for the fatty acid analysis. The lipids were saponified by the addition of potassium hydroxide methanol; the methyl esterification was carried out with boron (tri)fluoride–methanol solution; and *n*-hexane was used to extract the fatty acid methyl esters. After filtration, the sample was injected into a labeled sample vial and placed into the injection system of the gas chromatograph for detection. The results were shown as % total fatty acid (%TFA).

### 2.5. Muscle Texture and Serum Biochemistry Parameters

The index of muscle texture measurement included hardness, adhesiveness, cohesiveness, springiness, gumminess, and chewiness. Two additional experimental fish per tank were collected and the fillets were measured with a TMS-PRO (Food Technology Corporation, Sterling, VA, USA) texture analyzer equipped with a gravity sensor (25 N). The probe diameter was set to be 8 mm; test speed to be 30 mm/min; and deformation ratio to be 30%.

The concentration of total bile acids (TBA), malondialdehyde (MDA), protein carbonyls (PCO), glucose (Glu), total proteins (TP), total triacylglycerol (TG), total cholesterol (TC), low-density lipoprotein cholesterol (LDL-C), and high-density lipoprotein cholesterol (HDL-C) in this experiment were all measured using commercial kits (Nanjing Jiancheng, Nanjing, China) according to the users' manual.

### 2.6. The Real-Time Quantitative Polymerase Chain Reaction (RT-qPCR)

Total RNA from three tissue samples per tank (six samples per group) was extracted using the RNAiso Plus kit (TaKaRa, Dalian, China), and the RNA was reverse transcribed using Evo M-MLVRT and the gDNA Clean for qPCR Reverse Transcription Premix Kit (Ekorui Biotech Co., Ltd., Hunan, China). The SYBR Green Pro Taq HS premixed qPCR kit II (Acres Biotechnology Co., Ltd., Hunan, China) and a Roche fluorescence RT-qPCR instrument were used to perform the qPCR. According to previously established methods, two reference genes, *ef1α* and *β-actin*, were selected to calculate the mRNA expression of the target genes [42]. The primer sequences used are listed in Table 3.

The RT-qPCR system consisted of 5 *μ*L of SYBR Green Pro Taq HS Premix II, 4.4 *μ*L cDNA, and 0.3 *μ*L primer. The thermocycling conditions were as follows: first, hold at 95°C—30 s, and then perform 40 cycles under the conditions of “95°C—5 s, 57°C—30 s, 72°C—30 s.” A melting curve was plotted by warming from 65°C to 97°C at a rate of 6.4°C min^−1^. The relative mRNA expression of genes was expressed by the method of 2^−*ΔΔ*Ct^.

### 2.7. Statistical and Calculation Methods

SPSS 16.0 was used to perform the one-way ANOVA. Multiple comparisons were conducted using Tukey's test, and the calculations were presented as mean ± standard error. Significant differences were considered when *P* < 0.05. Regression analysis between evaluation parameters and the FO replacement level was conducted where necessary, also by SPSS 16.0.

## 3. Results

### 3.1. Growth and Feed Utilization

**Figure 1:**
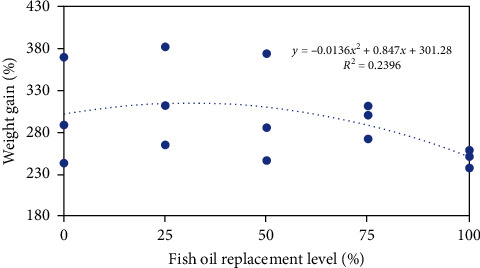
Regression analysis between fish oil replacement level and weight gain of tiger puffer.

Dietary FO replacement by PO did not have significant negative impacts on growth, feeding, and somatic indices. No significant differences were observed in survival, WG, FI, FCR, SGR, VSI, HSI, and K among all groups (*P* > 0.05, Table 4). However, the highest WG was observed in the 25PO group, but the lowest one was observed in the 100PO group, which was decreased by about 17.3% compared to the FO group (Table 4 and Figure 1). A quadratic regression was observed between weight gain and FO replacement level (Figure 1). According to this curve, the most suitable FO replacement level by PO was 31.1%.

### 3.2. Fish Body and Tissues Proximate Composition

The replacement of FO by PO in the diet had no significant influence (*P* > 0.05) on the proximate composition of the whole body (Table 5). There were cubic regression relationships between FO replacement level and some proximate composition parameters (muscle crude protein, muscle crude lipid, and liver crude lipid). The muscle crude protein content was significantly (*P* < 0.05) higher in the 50PO group than other groups except 75PO. The liver and muscle crude lipid contents were significantly higher in 25PO and 50PO compared to other groups (*P* < 0.05).

### 3.3. Muscle Texture

There was no significant difference in hardness, adhesiveness, cohesiveness, springiness, gumminess, and chewiness of muscle between the PO groups and the FO group (*P* > 0.05, Table 6).

### 3.4. Fatty Acid Composition in Fish Tissues

In the liver, the OA content increased linearly with increasing FO replacement levels, and the PA content increased significantly (*P* < 0.05, Table 7); however, both DHA and EPA content decreased significantly. Compared with the FO group, in the 100PO group, the liver PA content increased by about 19.9% and the OA content increased by about 73.7%; but the liver DHA content reduced by 56.4% and the EPA content reduced by 54.6% (Table 7 and Figure S2).

Similarly, in the intestine, the PA, OA, SFA, and MUFA contents increased with increasing FO replacement levels, and the DHA and EPA contents decreased (Table 8). The intestinal PA content in the 100PO group was increased by about 34.9%, and the OA content was increased by about 113% compared to the FO group; the intestinal DHA content in the 100PO group was decreased by 43.5% and the EPA content was decreased by 53.4% compared to the FO group (Table 8 and Figure S2).

Compared with liver and intestine, the replacement of FO by PO in the diet had less influence on the fatty acid composition of muscle, especially LC-PUFA. The muscle DHA, EPA, and arachidonic acid (ARA, 20 : 4n-6) contents were significantly reduced only in the 100PO group (Table 9, *P* < 0.05), and the n-3 LC-PUFA content of the 25PO group was even slightly higher compared to the control group. The muscle PA content in the 100PO group was almost the same as that of the FO group, but the OA content increased by about 54%. In the 100PO group, the DHA content decreased by 17.8% and the EPA content decreased by 18.5% compared to the FO group (Table 9 and Figure S2). Significant cubic regressions were observed between FO replacement levels and most liver and muscle fatty acid contents.

### 3.5. Serum Biochemical Parameters

Regarding the serum biochemical indices related to lipid metabolism, there were no significant differences in TG, TBA, Glu, PCO, and LDL-C concentration among groups (*P* > 0.05, Table 10). As the level of FO replacement increased, the TC concentration in serum first significantly increased (*P* < 0.05) and then decreased, with the highest value in the 50PO group. However, the highest HDL-C content was observed in the 75PO group. As the level of FO replacement increased, the serum MDA content was reduced, and the content in the 100PO group was significantly (*P* < 0.05) lower compared with the FO group. Significant cubic regressions were observed between FO replacement levels and the significantly influenced parameters mentioned above.

### 3.6. Gene Expression Involved in Lipid Metabolism

**Figure 2:**
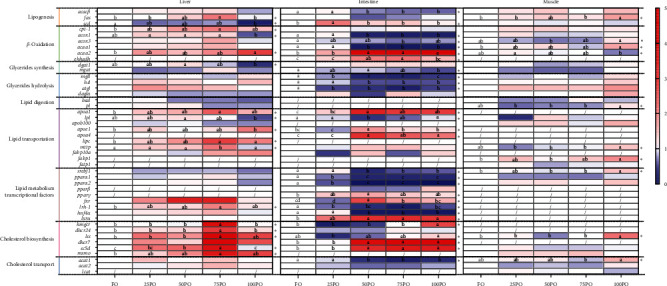
Relative mRNA expression of lipid metabolism genes in the liver and intestine of tiger puffer. For a certain gene, the mRNA expression in group FO was normalized to be 1. The significant regressions were marked with  ^*∗*^. For a specific gene in a specific tissue, blocks not sharing a same letter are significantly different (*P* < 0.05). Not detectable were marked with /.

In the liver, the replacement of FO by PO in diet, in particular, the 75PO group, significantly (*P* < 0.05) upregulated the gene expression involved in fatty acid *β*-oxidation, such as *cpt-1* and *acaa2* (Figure 2 and Table S1). The addition of PO had little effect on the gene expression involved in triglyceride synthesis and hydrolysis. In addition, the replacement of FO by PO in diet, particularly the 75PO group, significantly upregulated the expression of cholesterol biosynthetic genes, such as *hmgcr*, *dhcr24*, *lss*, *dhcr7*, *sc5d*, and *msmo*. The complete replacement of FO by PO significantly (*P* < 0.05) downregulated the *scd* expression (Figure 2 and Table S1).

In the intestine, the replacement of more than 50% FO by PO in diet, significantly (*P* < 0.05) downregulated the gene expression involved in peroxisomal fatty acid *β*-oxidation and triglyceride hydrolysis, such as *acox1*, *acaa1*, *mgll*, *hsl*, and *atgl*, while upregulated the gene expression involved in cholesterol synthesis (Figure 2 and Table S3).

In the muscle, only the complete replacement of FO significantly upregulated the expression of a few individual genes, such as *fas*, *acaa1*, *srebf1*, *fabp1*, and *lss*. However, there was no significant effect on the expression of most genes involved in lipid metabolism (Figure 2 and Table S2).

## 4. Discussion

In the present study, the complete replacement of FO by PO did not significantly inhibit the growth performance and feed efficiency of tiger puffer. This was similar to the results of previous research on Atlantic salmon [1], rainbow trout [22], Japanese seabass [23], Arctic charr [24], and greater amberjack [25], which showed that complete FO replacement with PO did not significantly influence the growth performance. However, a cubic regression was observed between FO replacement levels and fish weight gain. Partial FO replacement (25%) resulted in the highest weight gain and complete FO replacement still decreased the weight gain by 17.3%. In a longer feeding period, complete FO replacement may significantly reduce the growth. In previous research on other fish species, it was found that the FO replacement by PO above a certain level will significantly inhibit fish growth, such as what was observed on Indian mrigal (>25%) [28], Japanese flounder (>40%) [29], and Chu's Croaker (>80%) [30]. The discrepancy may be due to fatty acid utilization capacity of different fish species, feeding duration, water temperature, and interactions between nutrients.

Besides the growth, the n-3 LC-PUFA content in the muscle of commercial aquaculture fish species is a primary concern of human consumers [3, 13, 14, 43, 44]. The fatty acid composition in fish tissues usually closely reflects the dietary fatty acid compositions [2, 3, 36]. However, in this experiment, the FO replacement by PO, even up to the 75% level, only had a minor influence on muscle fatty acid composition. Compared with the FO control group, only in the 100PO group, the muscle DHA (from 18.0% to 14.8%) and EPA (from 4.96% to 4.04%) was obviously decreased. In the 25% replacement level, the muscle DHA content was even higher than the FO control group. This result supported the “n-3 LC-PUFA sparing effects” of MUFA and SFA and (mainly 18 : 1n-9 and 16 : 0) in PO [11, 12, 13]. Higher levels of MUFA and SFA may be preferentially *β*-oxidized for energy supply, and therefore spare the n-3 LC-PUFA to be used. In a previous study on Atlantic salmon, it was found that FO replacement by PO led to a linear decrease of EPA (from 5.70% to 1.90%), and a significant decrease of DHA (from 16.3% to 7.70%, a decrease of 52.8%) when FO was completely replaced [7]. The difference between Atlantic salmon and tiger puffer could be due to the fact that tiger puffer is a lean fish, having a much lower muscle lipid content than Atlantic salmon [36, 38, 39]. Higher polar lipid contents in leaner fish help to selectively deposit LC-PUFA such as DHA and EPA in the muscle [45, 46, 47, 48].

For farmed fish, a problem in the fish fillet could be the high contents of linoleic acid (LA, 18 : 2n-6), which could be harmful to human health, when high levels of vegetable oils are included in fish diets. In tiger puffer, when the lipid source changed from FO to soybean oil, the LA content in the dorsal muscle increased from 2.10% to 24.7% (%TFA) [32]. However, when the added marine FO was completely replaced by PO, the muscle LA content merely increased from 10.6% to 15.8% (%TFA). In this consideration, the use of PO could be more beneficial to human consumers.

Compared with muscle, the tiger puffer liver was more susceptible to the dietary lipid effects. In particular, the liver of tiger puffer is an important tissue for lipid storage, making it more closely reflect the dietary fatty acid profile than muscle. In the 100PO group, the liver PA content increased by about 19.9% and the OA content increased by about 73.7%. In contrast, the liver DHA content decreased by 56.4% and the EPA content decreased by 54.6%. The fact that EPA and DHA are less selectively deposited in lipid storage organ may explain the more pronounced reduction of DHA and EPA in the liver in response to dietary PO. Compared with the composition in liver, the intestinal fatty acid composition was more drastically affected by diets. The intestinal PA content increased by about 34.9% and the OA content increased by about 113% in the PO100 group compared with the control group; the intestinal DHA content was lowered by 43.5% and EPA content by 53.4%. The intestinal fatty acid composition may more closely reflect the newly absorbed fatty acids from the feed intake. In summary, the degree of changes in tissue fatty acids due to FO replacement by PO ranked as follows: intestine > liver > muscle.

Consistent with the fatty acid composition, at the transcription level, the lipid metabolism was also mostly affected in the intestine. The active lipid metabolism in fish intestine has been reported in previous studies [49, 50]. The PO downregulated the lipid synthesis, peroxisomal fatty acid *β*-oxidation, and triglyceride hydrolysis. The downregulation of peroxisomal *β*-oxidation of fatty acids could be due to the reduction of dietary very long-chain PUFA, which are preferential substrate of peroxisomal *β*-oxidation [51]. It was not expected that the PO downregulated both the lipid synthesis and triglyceride hydrolysis, which was not consistent with the tissue lipid content results. In general, FO replacement at high levels usually leads to an increase in the lipid content of fish tissues [3]. The inhibition of lipid synthesis and triglyceride hydrolysis could be regulated by feedback mechanisms.

In the liver, the supplementation of PO significantly upregulated the expression of the key mitochondrial *β*-oxidation gene *cpt-1*. High levels of PO also upregulated the gene expression associated with fatty acid *β*-oxidation, such as *acox1* and *acaa2*. This supported the n-3 LC-PUFA sparing effects of abundant MUFA and SFA in PO. In particular, the mitochondrial *β*-oxidation limiting enzyme, *cpt-1*, had a strong affinity for C10-18 fatty acids [52]. A study on yellow catfish (*Pelteobagrus fulvidraco*) also demonstrated that palmitic acid could strengthen liver health by activating mitochondrial *β*-oxidation [53].

Another noteworthy result in the gene expression was that PO stimulated the cholesterol biosynthesis in both liver and intestine. This regulation was probably also via a feedback mechanism, considering that PO contained much lower cholesterol than FO. This stimulation even resulted in higher final cholesterol levels (TC and HDL-C) in the serum. The results also indicate that *dhcr7* and *sc5d* could be key genes involved in cholesterol synthesis of fish. This was similar to what was observed in our previous studies [54].

In the muscle, only the complete replacement of FO significantly upregulated the expression of a few individual genes, such as *fas*, *acaa1*, *srebf1*, *fabp1*, and *lss*. However, there was no significant effect on the gene expression involved in lipid metabolism. This could be due to the fact that muscle is not a key tissue for lipid metabolism in tiger puffer.

In this study, the gene expression regarding to lipid peroxidation was not analyzed. However, the serum biochemical parameters clearly indicate that the FO replacement by PO reduced the serum MDA concentration, indicating a lower lipid peroxidation level. Similar results were found in Japanese seabass [55], Chu's Croaker [30], and tiger puffer [36, 37, 38]. The LC-PUFA are easily peroxidized and thus reduced LC-PUFA content in both diets and fish tissues may contribute to the lowered MDA content in the PO groups.

## 5. Conclusions

The FO replacement by PO did not have a significant adverse effect on the growth performance and feed utilization of tiger puffer, but there was a decreasing trend (reduced by 17.3%) in weight gain in the complete replacement group. The supplementation of PO also had no adverse effects on proximate composition and muscle texture. The change in muscle DHA and EPA contents was acceptable up to the 75% FO replacement level, but the changes in liver and intestine fatty acid profile were more drastic in response to diets. In the intestine, the replacement of more than 50% FO by PO in diets significantly downregulated the gene expression associated with peroxisomal fatty acid *β*-oxidation and triglyceride hydrolysis. In the liver, the replacement of FO with PO significantly upregulated the gene expression involved in fatty acid *β*-oxidation. In both liver and intestine, high levels of PO upregulated the expression of cholesterol biosynthetic genes. The replacement of FO by PO reduced the serum MDA concentration. Similar studies through longer term feeding duration are recommended in the future.

## Figures and Tables

**Table 1:** Formulation and proximate composition of the diets used in this experiment (% dry matter).

Ingredient	FO	25PO	50PO	75PO	100PO
Fishmeal^a^	40.0	40.0	40.0	40.0	40.0
Corn gluten meal^a^	10.0	10.0	10.0	10.0	10.0
Soybean meal^a^	10.0	10.0	10.0	10.0	10.0
Wheat meal^a^	22.68	22.68	22.68	22.68	22.68
Brewer's yeast^a^	5.00	5.00	5.00	5.00	5.00
Mineral premix^b^	0.50	0.50	0.50	0.50	0.50
Vitamin premix^b^	1.00	1.00	1.00	1.00	1.00
Monocalcium phosphate	1.00	1.00	1.00	1.00	1.00
L-ascorbyl-2-polyphosphate	0.20	0.20	0.20	0.20	0.20
Choline chloride	0.20	0.20	0.20	0.20	0.20
Betaine	0.30	0.30	0.30	0.30	0.30
Ethoxyquin	0.02	0.02	0.02	0.02	0.02
Calcium propionic	0.10	0.10	0.10	0.10	0.10
Soya lecithin	1.00	1.00	1.00	1.00	1.00
Fish oil	8.00	6.00	4.00	2.00	0.00
Palm oil	0.00	2.00	4.00	6.00	8.00
Total	100	100	100	100	100
Proximate composition					
Moisture	5.32	5.28	5.42	5.87	5.95
Crude protein	45.4	45.6	45.7	45.6	45.6
Crude lipid	11.4	11.4	11.4	11.2	10.8
Ash	10.0	9.80	9.80	9.80	9.90

^a^The crude protein contents of fishmeal, corn gluten meal, soybean meal, wheat meal, and brewer's yeast were 71.04%, 62.28%, 53.32%, 12.61%, and 48.83% (% dry matter), respectively. The crude lipid contents of them were 8.38%, 1.07%, 1.37%, 0.85% and 0.52% (% dry matter), respectively. All these ingredients were purchased from Qingdao Surgreen Bioengineering Co., Ltd. (Qingdao, China). ^b^Vitamin premix and mineral premix, specially designed for marine carnivorous fish, were provided by Qingdao Master Biotech Co., Ltd. (Qingdao, China).

**Table 2:** The fatty acid composition of the experimental diets and oil sources (% total fatty acids).

Fatty acid	Fish oil	Palm oil	FO	25PO	50PO	75PO	100PO
14 : 0	4.62	0.82	5.39	4.85	3.97	3.01	2.36
16 : 0	18.09	38.3	21.1	23.63	26.8	30.1	33.4
18 : 0	4.18	4.15	4.40	4.30	4.31	4.24	4.29
SFA	26.9	43.3	30.9	32.8	35.0	37.3	40.0
16 : 1n-7	4.95	0.15	4.82	4.10	3.32	2.41	1.72
18 : 1n-9	15.27	43.5	14.5	18.1	22.4	26.4	31.9
MUFA	20.22	43.7	19.4	22.2	25.7	28.8	33.6
18 : 2n-6	10.1	11.3	12.8	12.9	13.1	13.3	13.4
20 : 2n-6	0.32	0.01	0.31	0.25	0.22	0.16	0.46
n-6 PUFA	10.4	11.3	13.1	13.2	13.4	13.5	13.9
18 : 3n-3	2.12	0.15	1.88	1.57	1.29	0.92	0.63
20 : 5n-3	6.32	0.00	6.87	6.01	5.01	4.10	3.07
22 : 6n-3	11.1	0.00	10.0	8.67	6.90	5.40	3.70
n-3 PUFA	19.5	0.15	18.7	16.3	13.2	10.4	7.43
DHA/EPA	1.75	1.09	1.46	1.44	1.38	1.32	1.21

SFA, saturated fatty acid; MFUA, monounsaturated fatty acid; and PUFA, polyunsaturated fatty acid.

**Table 3:** Sequences of the primers for lipid metabolism genes studied in this work.

Primer	Sequence (5′-3′)	GenBank reference	PL (bp)
*Lipid metabolism gene*			
Lipogenesis			
* acacβ*-F	GAAAGGTTTGCTGTGCGACTA	XM_011615767.1	154
* acacβ*-R	TTACATCAGCGACCATTTCAGT		
* fas*-F	CTTTGCCGCTGTCATTCG	XM_011619859.1	78
* fas*-R	TGTCTCAACCCATTTGTAGTCG		
* scd*-F	ACCTCATGTGCTACTTGGGC	XM_011614011.2	118
* scd*-R	GCAGTGCAGACTTAGCCACT		
*β*-oxidation			
* cpt*-1-F	GGGGTTTGTGGTCAAGTTAGG	XM_011607269.1	186
* cpt*-1-R	ATAGATCCGTGGCGCTCAT		
* vlcs*-F	CGCTGTTCTTGGTGTTGGAC	XM_003969871.3	276
* vlcs*-R	GAGATTTGCTGCGGATGTTG		
* acox1*-F	GCACGGCATCGCAAGTAAC	XM_029850253.1	145
* acox1*-R	GAGATCGAAGGCATCCACC		
* acox3*-F	GACTGTGGCTATCCGCTTCT	XM_029839734.1	214
* acox3*-R	TTCCTGTCGGTCACTCTTGT		
* ehhadh*-F	GGCACAATGGGAAGAGGCATT	XM_003961946.3	185
* ehhadh*-R	TGGACGGTTTCGCTGTAGGTA		
* acaa1*-F	GGACAACAGCAAAGCAAGAG	XM_029849183.1	110
* acaa1*-R	ACCAGAAAAAACAGCCAAAA		
* acaa2*-F	ACGGGGGTGTTTTGAAGGA	XM_003975006.3	159
* acaa2*-R	CATGACGGGCAATGTAGGG		
* dgat1*-F	TGGTTTGTGAGCCGTTTCC	XM_003969352.2	185
* dgat1*-R	CTGGCATTCGTTTGACTTCG		
* mgat2a*-F	AAAGGCTTCATTAAATTGGC	XM_003978609.3	223
* mgat2a*-R	TGATGGCTTGTCTGTAGGG		
Hydrolysis of glycerides			
* atgl*-F	CCAACCTCTACAGGGTCTCA	XM_003967696.3	119
* atgl*-R	GTTTAGCAGCCCGTTCTTC		
* daglα*-F	CTGTTGGTGGAGTTGGTGTATG	XM_011610175.1	72
* daglα*-R	ATCAGAGCACGGCTGGTAAT		
* hsl*-F	CTCTTGCTATCGGTCTTGTGG	XM_011621066.1	113
* hsl*-R	TTCTGGGTCAATGGCATACTT		
* mgll*-F	CCATCCAGTCAAAGTGGGTCT	XM_003963030.2	110
* mgll*-R	CATCAGCTGCATGCCGAA		
Lipid digestion			
* bsal*-F	TTGAAGATGACTGACCCCGA	XM_003978375.2	162
* bsal*-R	GATGTCTGCTGCGTTGTGAA		
* lp*-F	CGTTTTCTCCTGTTCACCC	XM_029832009.1	97
* lp*-R	GACTCGTCCTCATCCCACT		
Lipid transport			
* lpl*-F	AGGGTCCACATCCGCAAA	NM_001305600.1	157
* lpl*-R	GTTTCTCCTTGCGGCTCAT		
* lipc*-F	GCGGCTTCAACAGCAGTAA	XM_011610357.1	215
* lipc*-R	GAGGTGCGCTATGTCTTTCC		
* fabp1*-F	CCATCGGTCTCCCTGATGAAG	XM_003974807.3	121
* fabp1*-R	TTGACCGTTACCTTCGGTCC		
* fabp10a*-F	CTGTGACCAACTCCTTTACCAT	XM_003965635.3	150
* fabp10a*-R	TCTCTCCACCTTTGAGCTCCTG		
* fatp1*-F	ATTGCAGACACCACAGGGAG	XM_003964742.3	219
* fatp1*-R	ATATCGTGACGCTCGTGCAT		
* apoa1*-F	CGATGACGCCGAGTACAAA	AB183289.1	104
* apoa1*-R	CGGTTATGGGAGAAACGCTA		
* apoa4*-F	TGCTTTCTGGGACTATGTTGC	NM_001078591.1	124
* apoa4*-R	GTTGACTTTGTCGGCACTCTC		
* apob100*-F	AGGGACATAGTCAAACCAAGGA	XM_011619944.1	127
* apob100*-R	AGAACACGAAGGCTGGACAC		
* apoe1*-F	TATTCAGACCCGCACCTCA	NM_001078592.1	201
* apoe1*-R	ATTTCCTCCATCTTGTCCTCC		
* mttp*-F	ATGCTAAGGGTCTGGTTCTGC	XM_011612378.1	124
* mttp*-R	ATGTCAGTGCTGCCGATCTT		
Lipid metabolism-related transcriptional factors			
* srebf1*-F	TTTCAGCATCCCACCTTCC	XM_011603881.1	158
* srebf1*-R	GGTGAACCGTGAGGACAACTA		
* pparα1*-F	TCAGTAGTTTATGGGTTGGTGG	NM_001097630.1	119
* pparα1*-R	GCGTGGACTCCGTAGTGGTA		
* pparα2*-F	CCAGAAGAAGAACCGCAACA	NM_001097629.1	149
* pparα2*-R	CCTCTTTCTCCACCATCTTGTT		
* pparβ*-F	AGCTGGAATACGACCGATGT	AB275887.1	249
* pparβ*-R	TCTTCAGGTAGGCGGAGTTG		
* pparγ*-F	CGCTGTCCCGACATCTGTAT	NM_001097627.1	146
* pparγ*-R	GAACTGCTCGCCTTCCATT		
* fxr*-F	GTGAACGACCACAAGTTTACCC	XM_003967283.2	166
* fxr*-R	AGACCAACAGATTACACCGGAT		
* lxrα*-F	GTGACGCACCACTAACAGCA	XM_011609917.1	191
* lxrα*-R	CTGACAACACCGAGCAAGACT		
* hnf4α*-F	GAGCCACGGGCAAACACTA	XM_011619034.1	199
* hnf4α*-R	AGGGTCCTACCTTCTTTCTTCAT		
* lrh-1*-F	CGCTGACATGCTGCCTAAA	XM_003974281.2	140
* lrh-1*-R	TCTCGTCCAAGTCTTCGTCAT		
Cholesterol acid biosynthesis			
* hmgcr*-F	GCTGCTGGCAATCAAGTACAT	XM_003974466.2	143
* hmgcr*-R	AAACATACAACTCCTTCCTACAGC		
* msmo*-F	GGACAAACCAGAGACCTGGG	XM_003972423.3	138
* msmo*-R	GAGTCCCAGTCGTAGGGGAT		
* lss*-F	GGGCCGCTTCTCTCATCTTT	XM_003962203.3	71
* lss*-R	ACGTTGAGGCCTTTACAGCA		
* dhcr24*-F	CAAAATCTGCAAGTCGGCCC	XM_003973858.3	145
* dhcr24*-R	TTTGGTGGCGCCAATGAAAG		
* dhcr7*-F	AAGACTGGAAGCGCTACACC	XM_029846564.1	101
* dhcr7*-R	CTGATGCTCTCAGCCCTCAC		
* sc5d*-F1	CCGACCACTATGTCCTCACG	XM_011611310.2	141
* sc5d*-R1	AGTAACTGAACGACGCCAGG		
Cholesterol transport related			
* acat1*-F	TTGGGTTCGGTTGTGAAT	XM_003976083.3	114
* acat1*-R	GAGGCAGATGGAGGTGGT		
* acat2*-F	ACGCCTCAGGTATGAACGAC	XM_003971888.3	186
* acat2*-R	GTTTTACGCCACGCTTCTCG		
* lcat*-F	TGACTATGAGGACGGGTGGT	XM_003977753.3	78
* lcat*-R	GTGTTGTCCCCATCAGCGTA		
Reference gene			
*β-actin*-F	GAGAGGGAAATCGTGCGTGA	XM_003964421.3	186
*β-actin*-R	GAAGGATGGCTGGAAGAGGG		
*ef1α*-F	TTGGAGGCATTGGAACTGT	NM_001037873.1	86
*ef1α*-R	GTTGACGGGAGCAAAGGT		

*acacβ*, acetyl-CoA carboxylase beta; *fas*, fatty acid synthase; *scd*, stearoyl-CoA desaturase; *cpt-1*, carnitine O-palmitoyltransferase-1; *acox*, acyl-CoA oxidase; *acaa*, acetyl-CoA acyltransferase; *dgat1*, diacylglycerol O-acyltransferase 1; *mgat2a*, 2-acylglycerol O-acyltransferase 2-A-, like; *atgl*, adipose triglyceride lipase; *daglα*, diacylglycerol lipase, alpha; *hsl*, hormone-sensitive lipase; *mgll*, monoglyceride lipase; *bsal*, bile acid activated lipase; *lp*, inactive pancreatic lipase-related protein 1-like; *lpl*, lipoprotein lipase; *lipc*, lipase, hepatic; *fabp*, fatty acid binding protein; *fatp*, fatty acid transport protein; *apo*, apolipoprotein; *srebf1*, sterol regulatory element binding transcription factor 1; *ppar*, peroxisome proliferator-activated receptor; *fxr*, farnesoid X receptor; *lxrα*, liver X receptor alpha; *hnf4α*, hepatocyte nuclear factor 4, alpha; *lrh-1*, liver receptor homolog-1; *hmgcr*, 3-hydroxy-3-methylglutaryl-CoA reductase; *msmo*, methylsterol monooxygenase; *lss*, lanosterol synthase (2,3-oxidosqualene-lanosterol cyclase); *dhcr*, dehydrocholesterol reductase; *sc5d*, sterol-C5-desaturase; *acat*, cholesterol acyltransferase; *lcat*, lecithin cholesterol acyl transferase; and PL, sequence length.

**Table 4:** Growth performance and somatic parameters of experimental tiger puffer at week 8 (*n* = 3).

Parameters	FO	25PO	50PO	75PO	100PO	Regression			
						Model	Equation	*R* ^2^	*P*
IBW (g)	15.0 ± 0.05	15.0 ± 0.05	14.9 ± 0.04	15.0 ± 0.04	14.9 ± 0.04	Cubic	*y* = −0.563*x*^3^ + 1.0667*x*^2^−0.6204*x* + 15.051	0.298	0.256
FBW (g)	60.2 ± 5.40	62.7 ± 4.92	59.8 ± 5.63	59.0 ± 1.85	52.0 ± 0.91	Quadratic	*y* = −19.469*x*^2^ + 11.461*x* + 60.315	0.250	0.178
Survival (%)	90.0 ± 0.00	82.2 ± 4.45	83.3 ± 6.94	84.4 ± 6.19	87.8 ± 7.78	Cubic	*y* = −35.556*x*^3^ + 78.73*x*^2^−45.397*x* + 89.841	0.170	0.729
WG (%)	300 ± 37.2	319 ± 34.1	301 ± 37.8	294 ± 11.8	248 ± 6.22	Quadratic	*y* = −136.02*x*^2^ + 84.701*x* + 301.28	0.240	0.193
FI (%/day)	3.01 ± 0.18	3.05 ± 0.21	3.15 ± 0.41	3.14 ± 0.16	3.13 ± 0.23	Quadratic	*y* = −0.2307*x*^2^ + 0.3601*x* + 3.0005	0.021	0.882
FCR	1.51 ± 0.16	1.49 ± 0.14	1.58 ± 0.27	1.58 ± 0.10	1.67 ± 0.12	Quadratic	*y* = 0.1339*x*^2^ + 0.0303*x* + 1.4975	0.060	0.691
SGR (%/day)	2.34 ± 0.15	2.42 ± 0.14	2.34 ± 0.16	2.32 ± 0.05	2.12 ± 0.03	Quadratic	*y* = −0.5954*x*^2^ + 0.3811*x* + 2.3391	0.249	0.180
HSI (%)	15.8 ± 0.54	15.8 ± 0.55	15.5 ± 0.69	17.1 ± 0.54	15.5 ± 0.53	Cubic	*y* = −0.2391*x*^3^ + 2.0643*x*^2^ − 5.0662*x* + 19.183	0.097	0.441
VSI (%)	11.9 ± 0.14	11.4 ± 0.32	11.4 ± 0.54	12.1 ± 0.48	11.4 ± 0.13	Cubic	*y* = −0.1547*x*^3^ + 1.4035*x*^2^ − 3.7518*x* + 14.431	0.205	0.451
K (g/cm^3^)	2.69 ± 0.08	2.68 ± 0.17	2.72 ± 0.05	2.87 ± 0.11	2.72 ± 0.09	Cubic	*y* = −0.0392*x*^3^ + 0.3474*x*^2^ − 0.8809*x* + 3.2594	0.211	0.300

Data in a same row not sharing a same superscript letter are significantly different (*P* < 0.05). IBW, Initial body weight; FBW, final body weight; WG, weight gain; FCR, feed conversion ratio; SGR, specific growth ratio; HSI, hepatosomatic index; VSI, viscerosomatic index; K, condition factor; survival (%) = final fish number/initial fish number × 100; weight gain (WG, %) = (FBW − IBW)/IBW × 100; feed intake (FI, %) = feed dry weight/(experimental days × (IBW + FBW)/2) × 100; feed conversion ratio (FCR) = feed intake/weight gain; viscerosomatic index (VSI, %) = wet viscera weight/fish body weight × 100; hepatosomatic index (HSI, %) = wet liver weight/fish body weight × 100; and condition factor (K) = body weight/(body length^3^) × 100.

**Table 5:** Whole-body and tissue proximate composition of experimental tiger puffer (% wet weight).

Parameters	FO	25PO	50PO	75PO	100PO	Regression			
						Model	Equation	*R* ^2^	*P*
Whole body									
Crude protein	15.6 ± 0.13	15.4 ± 0.33	15.8 ± 0.17	15.3 ± 0.46	15.9 ± 0.16	Cubic	*y* = −0.0456*x*^3^ + 0.407*x*^2^ − 0.9353*x* + 16.149	0.146	0.406
Crude lipid	5.73 ± 0.17	5.81 ± 0.22	5.70 ± 0.20	6.03 ± 0.23	5.71 ± 0.12	Cubic	*y* = −0.0378*x*^3^ + 0.3138*x*^2^ − 0.7175*x* + 6.1986	0.033	0.828
Moisture	74.8 ± 0.20	74.5 ± 0.45	75.5 ± 0.43	74.2 ± 0.63	74.8 ± 0.33	Cubic	*y* = 0.0578*x*^3^ − 0.5274*x*^2^ + 1.3613*x* + 73.821	0.029	0.927
Ash	2.33 ± 0.06	2.21 ± 0.05	2.25 ± 0.09	2.51 ± 0.04	2.27 ± 0.11	Cubic	*y* = −0.0546*x*^3^ + 0.4902*x*^2^ − 1.263*x* + 3.1688	0.407	0.112
Muscle									
Crude protein	17.6 ± 0.01^bc^	17.6 ± 0.22^c^	18.9 ± 0.10^a^	18.6 ± 0.01^ab^	17.7 ± 0.34^bc^	**Cubic**	***y* = −0.1576*x*** **^3^** ** + 1.1799*x*** **^2^ ** **− ** **2.1725*x* + 18.671**	**0.695**	**0.004**
Crude lipid	2.69 ± 0.04^b^	2.97 ± 0.02^a^	3.00 ± 0.06^a^	2.73 ± 0.08^b^	2.72 ± 0.02^b^	**Cubic**	***y* = 0.0441*x*** **^3^ ** **−** ** ** **0.4598*x*** **^2^** ** + 1.4028*x* + 1.6875**	**0.725**	**0.002**
Moisture	79.7 ± 0.14	79.6 ± 0.08	78.4 ± 0.68	78.8 ± 0.58	79.4 ± 0.18	Cubic	*y* = 0.1076*x*^3^ − 0.7661x^2^ + 1.1895x + 79.188	0.316	0.226
Liver									
Crude protein	5.51 ± 0.09	5.85 ± 0.21	5.32 ± 0.15	5.88 ± 0.16	5.67 ± 0.19	Cubic	y = −0.0022x^3^ + 0.0375*x*^2^ − 0.1503*x* + 5.81	0.011	0.989
Crude lipid	51.2 ± 0.51^b^	55.4 ± 0.91^a^	55.7 ± 1.08^a^	47.7 ± 0.83^b^	48.3 ± 0.56^b^	**Cubic**	***y* = 1.0328*x*** **^3^ ** **−** ** ** **10.401*x*** **^2^** ** + 29.667*x* + 30.61**	**0.797**	**0.000**

Data in a same row not sharing a same superscript letter are significantly different (*P* < 0.05). The significant regressions were marked in bold.

**Table 6:** Effects of fish oil replacement by palm oil on the muscle texture of experimental tiger puffer.

Parameters	FO	25PO	50PO	75PO	100PO	Regression			
						Model	Equation	*R* ^2^	*P*
Hardness (N)	2.71 ± 0.69	3.26 ± 0.67	2.99 ± 0.55	3.72 ± 0.63	2.93 ± 0.34	Cubic	*y* = −0.0754*x*^3^ + 0.4998*x*^2^ − 0.4348*x* + 2.174	0.390	0.043
Adhesiveness (mJ)	0.03 ± 0.003	0.03 ± 0.002	0.03 ± 0.003	0.03 ± 0.002	0.03 ± 0.003	Cubic	*y* = −0.0003*x*^3^ + 0.0017*x*^2^ − 0.0014*x* + 0.0317	0.021	0.904
Cohesiveness (ratio)	0.41 ± 0.02	0.43 ± 0.04	0.39 ± 0.05	0.39 ± 0.04	0.41 ± 0.02	Cubic	*y* = 0.0056*x*^3^ − 0.0475*x*^2^ + 0.1136*x* + 0.3353	0.017	0.929
Springiness (mm)	1.29 ± 0.14	1.48 ± 0.09	1.44 ± 0.24	1.42 ± 0.09	1.38 ± 0.13	Cubic	*y* = 0.0035*x*^3^ − 0.0762*x*^2^ + 0.3517*x* + 0.9908	0.184	0.370
Gumminess (N)	1.14 ± 0.35	1.42 ± 0.37	1.26 ± 0.33	1.41 ± 0.24	1.17 ± 0.12	Cubic	*y* = −0.0374*x*^3^ + 0.2757*x*^2^ − 0.4115*x* + 1.0016	0.420	0.038
Chewiness (mJ)	1.03 ± 0.17	1.49 ± 0.21	1.9 ± 0.18	2.07 ± 0.38	1.36 ± 0.13	Cubic	*y* = −0.0698*x*^3^ + 0.4433*x*^2^ − 0.4109*x* + 1.0706	0.483	0.017

**Table 7:** The fatty acid composition in the liver of juvenile tiger puffer (% TFA).

Fatty acid	FO	25PO	50PO	75PO	100PO	Regression			
						Model	Equation	*R* ^2^	*P*
14 : 0	2.66 ± 0.05^a^	2.44 ± 0.06^ab^	2.22 ± 0.10^bc^	1.89 ± 0.15^c^	1.47 ± 0.01^d^	**Cubic**	***y* = −0.0076*x*** ^**3 **^ **+ 0.031*x*** ^**2**^ ** − 0.2494*x* + 2.8863**	**0.917**	**0.000**
16 : 0	20.6 ± 0.13^d^	21.5 ± 0.40^cd^	22.7 ± 0.32^bc^	23.5 ± 0.37^ab^	24.7 ± 0.22^a^	**Cubic**	***y* = −0.004*x*** ^**3 **^ **+ 0.0527*x*** ^**2**^ ** + 0.8177*x* + 19.736**	**0.914**	**0.000**
18 : 0	7.21 ± 0.40	7.21 ± 0.08	7.03 ± 0.23	7.57 ± 0.16	7.63 ± 0.25	Cubic	*y* = −0.025*x*^3^ + 0.2846*x*^2^ − 0.8277*x* + 7.8097	0.229	0.395
20 : 0	0.34 ± 0.02^a^	0.31 ± 0.00^ab^	0.28 ± 0.00^b^	0.27 ± 0.01^bc^	0.22 ± 0.00^c^	**Cubic**	***y* = −0.0024*x*** ^**3 **^ **+ 0.0197*x*** ^**2 **^ **− 0.0739*x* + 0.3947**	**0.860**	**0.000**
SFA	30.8 ± 0.49^c^	31.4 ± 0.46^c^	32.2 ± 0.15^bc^	33.2 ± 0.36^ab^	34.0 ± 0.17^a^	**Cubic**	***y* = −0.039*x*** ^**3 **^ **+ 0.3881*x*** ^**2**^ ** − 0.3333*x* + 30.827**	**0.839**	**0.000**
14 : 1n-5	0.47 ± 0.01^a^	0.42 ± 0.01^b^	0.35 ± 0.02^c^	0.29 ± 0.01^d^	0.20 ± 0.01^e^	**Cubic**	***y* = −0.0001*x*** ^**3**^ ** − 0.0028*x*** ^**2**^ ** − 0.044*x* + 0.5139**	**0.974**	**0.000**
16 : 1n-7	6.08 ± 0.21^a^	5.83 ± 0.06^ab^	5.43 ± 0.12^b^	4.65 ± 0.08^c^	3.80 ± 0.03^d^	**Cubic**	***y* = 0.0059*x*** ^**3 **^ **− 0.1655*x*** ^**2**^ ** + 0.242*x* + 5.9872**	**0.961**	**0.000**
18 : 1n-9	19.4 ± 0.09^e^	22.5 ± 0.46^d^	26.0 ± 0.41^c^	29.1 ± 0.38^b^	33.7 ± 0.50^a^	**Cubic**	***y* = 0.0928*x*** ^**3**^ ** − 0.6459*x*** ^**2**^ ** + 4.5683*x* + 15.36**	**0.987**	**0.000**
22 : 1n-9	0.17 ± 0.01^a^	0.16 ± 0.01^a^	0.14 ± 0.01^ab^	0.12 ± 0.00^b^	0.11 ± 0.01^b^	**Cubic**	***y* = 0.0025*x*** ^**3**^ ** − 0.0229*x*** ^**2**^ ** + 0.0436*x* + 0.1461**	**0.817**	**0.000**
MUFA	26.1 ± 0.30^e^	28.9 ± 0.45^d^	31.9 ± 0.30^c^	34.2 ± 0.45^b^	37.8 ± 0.46^a^	**Cubic**	***y* = 0.1011*x*** ^**3**^ ** − 0.8371*x*** ^**2**^ ** + 4.8098*x* + 22.007**	**0.979**	**0.000**
18 : 2n-6	10.3 ± 0.04	10.6 ± 0.26	10.4 ± 0.23	10.4 ± 0.11	10.7 ± 0.11	Cubic	*y* = 0.0802*x*^3^ − 0.704*x*^2^ + 1.8515*x* + 9.0511	0.308	0.238
18 : 3n-6	0.44 ± 0.01^a^	0.38 ± 0.02^b^	0.28 ± 0.01^c^	0.18 ± 0.01^d^	0.08 ± 0.00^e^	**Cubic**	***y* = 0.0034*x*** ^**3**^ ** − 0.0363*x*** ^**2**^ ** + 0.022*x* + 0.453**	**0.985**	**0.000**
20 : 2n-6	0.57 ± 0.02^ab^	0.58 ± 0.02^a^	0.53 ± 0.01^ab^	0.51 ± 0.01^ab^	0.49 ± 0.02^b^	**Cubic**	***y* = 0.0053*x*** ^**3**^ ** − 0.05*x*** ^**2**^ ** + 0.116*x* + 0.5005**	**0.640**	**0.009**
20 : 3n-6	0.89 ± 0.01^a^	0.75 ± 0.01^b^	0.57 ± 0.03^c^	0.41 ± 0.06^d^	0.14 ± 0.00^e^	**Cubic**	***y* = −0.0069*x*** ^**3**^ ** + 0.045*x*** ^**2**^ ** − 0.2424*x* + 1.0966**	**0.976**	**0.000**
20 : 4n-6	0.09 ± 0.01^ab^	0.10 ± 0.01^a^	0.07 ± 0.00^bc^	0.07 ± 0.00^c^	0.07 ± 0.01^bc^	**Cubic**	***y* = 0.0034*x*** ^**3**^ ** − 0.0296*x*** ^**2**^ ** + 0.0657*x* + 0.0555**	**0.669**	**0.005**
n-6 PUFA	12.3 ± 0.05^ab^	12.4 ± 0.30^a^	11.8 ± 0.23^ab^	11.6 ± 0.15^b^	11.5 ± 0.13^b^	**Cubic**	***y* = 0.0853*x*** ^**3**^ ** − 0.7749*x*** ^**2**^ ** + 1.8128*x* + 11.157**	**0.636**	**0.009**
18 : 3n-3	1.29 ± 0.11^a^	1.24 ± 0.10^ab^	0.92 ± 0.02^bc^	0.79 ± 0.07^c^	0.61 ± 0.03^c^	**Cubic**	***y* = 0.0186*x*** ^**3**^ ** − ** **0.174*x*** ^**2**^ ** + 0.2957*x* + 1.1591**	**0.838**	**0.000**
20 : 3n-3	0.56 ± 0.07^a^	0.39 ± 0.03^ab^	0.35 ± 0.01^b^	0.30 ± 0.03^b^	0.23 ± 0.03^b^	**Cubic**	***y* = −0.0127*x*** ^**3**^ ** + 0.1258*x*** ^**2**^ ** − 0.4435*x* + 0.8838**	**0.805**	**0.000**
20 : 5n-3	4.52 ± 0.04^a^	3.72 ± 0.10^b^	3.36 ± 0.08^c^	2.71 ± 0.08^d^	2.05 ± 0.02^e^	**Cubic**	***y* = −0.0372*x*** ^**3**^ ** + 0.335*x*** ^**2**^ ** − 1.4737*x* + 5.6849**	**0.983**	**0.000**
22 : 5n-3	2.64 ± 0.11^a^	2.48 ± 0.15^a^	2.22 ± 0.04^ab^	2.01 ± 0.06^bc^	1.66 ± 0.05^c^	**Cubic**	***y* = −0.0026*x*** ^**3**^ **−0.0003*x*** ^**2**^ ** − 0.164*x* + 2.8137**	**0.873**	**0.000**
22 : 6n-3	10.1 ± 0.14^a^	8.20 ± 0.30^b^	7.20 ± 0.28^b^	6.00 ± 0.21^c^	4.40 ± 0.14^d^	**Cubic**	***y* = −0.1094*x*** ^**3**^ ** + 1.0169*x*** ^**2**^ ** − 4.1157*x* + 13.251**	**0.973**	**0.000**
n-3 PUFA	19.1 ± 0.08^a^	16.0 ± 0.59^b^	14.0 ± 0.30^c^	11.8 ± 0.28^d^	9.00 ± 0.17^e^	**Cubic**	***y* = −0.1433*x*** ^**3**^ ** + 1.3035*x*** ^**2**^ ** − 5.9012*x* + 23.793**	**0.982**	**0.000**
DHA/EPA	2.22 ± 0.02	2.19 ± 0.03	2.14 ± 0.11	2.22 ± 0.07	2.16 ± 0.06	Cubic	*y* = −0.0098*x*^3^ + 0.0939*x*^2^ − 0.2744*x* + 2.4194	0.056	0.882

Data in a same row not sharing the same superscript letter were significantly different (*P*  < 0.05). TFA, total fatty acid; SFA, saturated fatty acid; MUFA, monounsaturated fatty acid; PUFA, polyunsaturated fatty acid; and LC-PUFA, long-chain polyunsaturated fatty acids. The significant regressions were marked in bold.

**Table 8:** The fatty acid composition in the intestine of juvenile tiger puffer (% TFA, means).

Fatty acid	FO	25PO	50PO	75PO	100PO
14 : 0	3.17	2.82	2.25	1.90	1.35
16 : 0	19.2	21.7	23.7	25.5	25.9
17 : 0	0.56	0.48	0.38	0.33	0.26
18 : 0	5.71	5.86	5.90	5.56	5.92
20 : 0	0.35	0.33	0.28	0.23	0.18
22 : 0	0.16	0.14	0.13	0.11	0.09
SFA	29.1	31.4	32.6	33.7	33.7
16 : 1n-7	4.28	3.71	2.90	2.40	1.65
17 : 1n-7	0.24	0.21	0.16	0.15	0.07
18 : 1n-9	13.3	17.5	21.0	25.4	28.3
20 : 1n-9	0.05	0.04	0.04	0.03	0.03
22 : 1n-9	0.17	0.14	0.12	0.09	0.07
24 : 1n-9	0.38	0.33	0.31	0.23	0.18
MUFA	18.4	21.9	24.5	28.3	30.3
18 : 2n-6	12.6	13.0	13.5	13.9	14.9
18 : 3n-6	0.32	0.25	0.16	0.09	—
20 : 2n-6	0.41	0.52	0.50	0.41	0.44
20 : 3n-6	1.05	0.60	0.53	0.27	0.02
20 : 4n-6	0.21	0.19	0.18	0.16	0.16
n-6 PUFA	14.5	14.6	14.8	14.8	15.5
18 : 3n-3	1.50	1.25	0.96	0.78	0.55
20 : 3n-3	1.46	1.22	1.09	0.84	0.77
20 : 5n-3	5.54	4.58	3.85	3.09	2.58
22 : 5n-3	2.52	2.18	2.13	2.04	2.07
22 : 6n-3	14.5	12.0	10.9	9.09	8.19
n-3 PUFA	25.5	21.2	18.9	15.8	14.1
DHA/EPA	2.61	2.61	2.82	2.92	3.14

TFA, total fatty acid; SFA, saturated fatty acid; MUFA, monounsaturated fatty acid; PUFA, polyunsaturated fatty acid; and LC-PUFA, long-chain polyunsaturated fatty acids.

**Table 9:** The fatty acid composition in muscle of juvenile tiger puffer (% TFA).

Fatty acid	FO	25PO	50PO	75PO	100PO	Regression			
						Model	Equation	*R* ^2^	*P*
14 : 0	0.66 ± 0.02^a^	0.53 ± 0.01^b^	0.46 ± 0.02^bc^	0.37 ± 0.04^cd^	0.27 ± 0.01^d^	**Cubic**	***y* = −0.0053** ^**3**^ ** + 0.0508*x*** ^**2**^ ** − 0.2399*x* + 0.8557**	**0.945**	**0.000**
16 : 0	25.1 ± 0.66	24.9 ± 0.17	24.6 ± 0.38	24.5 ± 0.42	24.7 ± 0.04	Cubic	*y* = 0.0357*x*^3^ − 0.2513*x*^2^ + 0.305*x* + 24.964	0.125	0.675
17 : 0	0.43 ± 0.03^a^	0.32 ± 0.01^b^	0.26 ± 0.00^c^	0.22 ± 0.00^c^	0.15 ± 0.01^d^	**Cubic**	***y* = −0.0057*x*** ^**3**^ ** + 0.0594*x*** ^**2**^ ** − 0.2506*x* + 0.6295**	**0.961**	**0.000**
18 : 0	8.68 ± 0.05	8.71 ± 0.39	7.93 ± 0.14	8.08 ± 0.22	7.74 ± 0.16	Cubic	*y* = 0.0276*x*^3^ − 0.2347*x*^2^ + 0.317*x* + 8.6177	0.507	0.044
20 : 0	0.32 ± 0.01^a^	0.28 ± 0.01^a^	0.22 ± 0.01^b^	0.20 ± 0.01^b^	0.15 ± 0.00^c^	**Cubic**	***y* = −0.0006*x*** ^**3**^ ** + 0.0061*x*** ^**2**^ ** − 0.062*x* + 0.3797**	**0.939**	**0.000**
SFA	35.1 ± 0.69	34.7 ± 0.22	33.4 ± 0.46	33.3 ± 0.66	33.0 ± 0.15	**Cubic**	***y* = 0.0519*x*** ^**3**^ ** −** ^** **^ **0.3697*x*** ^**2**^ ** + 0.0694*x* + 35.447**	**0.575**	**0.002**
14 : 1n-5	1.66 ± 0.03	1.64 ± 0.01	1.64 ± 0.08	1.74 ± 0.09	1.74 ± 0.09	Cubic	*y* = −0.01*x*^3^ + 0.0996*x*^2^ − 0.2694*x* + 1.8463	0.153	0.592
16 : 1n-7	1.03 ± 0.02^a^	0.83 ± 0.04^ab^	0.83 ± 0.06^ab^	0.63 ± 0.07^bc^	0.45 ± 0.02^c^	**Cubic**	***y* = −0.016*x*** ^**3**^ ** + 0.1332*x*** ^**2**^ ** − 0.4478*x* + 1.3534**	**0.875**	**0.000**
18 : 1n-9	13.7 ± 0.26^d^	15.0 ± 0.27^cd^	16.3 ± 0.35^bc^	17.8 ± 0.09^b^	21.1 ± 1.00^a^	**Cubic**	***y* = 0.1505*x*** ^**3**^ ** − 1.0642*x*** ^**2**^ ** + 3.5556*x* + 11.069**	**0.926**	**0.000**
MUFA	16.4 ± 0.27^c^	17.5 ± 0.28^c^	18.8 ± 0.28^bc^	20.2 ± 0.08^b^	23.3 ± 1.07^a^	**Cubic**	***y* = 0.1245*x*** ^**3**^ ** − 0.8314*x*** ^**2**^ ** + 2.8384*x* + 14.269**	**0.910**	**0.000**
18 : 2n-6	10.6 ± 0.11^c^	10.9 ± 0.21^c^	13.0 ± 0.57^b^	13.5 ± 0.27^b^	15.8 ± 0.29^a^	**Cubic**	***y* = 0.0109*x*** ^**3**^ ** + 0.0776*x*** ^**2**^ ** + 0.5137*x* + 9.8542**	**0.911**	**0.000**
20 : 2n-6	0.55 ± 0.00^a^	0.52 ± 0.01^ab^	0.52 ± 0.01^ab^	0.49 ± 0.02^b^	0.50 ± 0.01^ab^	**Cubic**	***y* = 0.0013*x*** ^**3**^ ** − 0.0066*x*** ^**2**^ ** − 0.0129*x* + 0.5682**	**0.569**	**0.022**
20 : 3n-6	0.35 ± 0.02^a^	0.26 ± 0.02^ab^	0.14 ± 0.00^bc^	0.12 ± 0.06^bc^	0.08 ± 0.04^c^	**Cubic**	***y* = 0.002*x*** ^**3**^ ** − 0.0031*x*** ^**2**^ ** − 0.1095*x* + 0.4633**	**0.788**	**0.001**
20 : 4n-6	0.11 ± 0.00^a^	0.09 ± 0.00^ab^	0.09 ± 0.01^ab^	0.09 ± 0.01^ab^	0.08 ± 0.01^b^	**Cubic**	***y* = −0.0017*x*** ^**3**^ ** + 0.0175*x*** ^**2**^ ** − 0.0608*x* + 0.1569**	**0.634**	**0.009**
n-6 PUFA	11.8 ± 0.12^c^	11.9 ± 0.24^c^	13.8 ± 0.57^b^	14.8 ± 0.20^b^	17.0 ± 0.26^a^	**Cubic**	***y* = 0.0217*x*** ^**3**^ ** + 0.0087*x*** ^**2**^ ** + 0.4473*x* + 11.228**	**0.893**	**0.000**
18 : 3n-3	0.36 ± 0.01^a^	0.33 ± 0.01^ab^	0.38 ± 0.07^a^	0.26 ± 0.02^ab^	0.19 ± 0.01^b^	**Cubic**	***y* = −0.0023*x*** ^**3**^ ** + 0.0024*x*** ^**2**^ ** + 0.015*x* + 0.3333**	**0.581**	**0.019**
20 : 3n-3	1.83 ± 0.05^a^	1.73 ± 0.03^a^	1.51 ± 0.08^b^	1.32 ± 0.02^b^	1.10 ± 0.02^c^	**Cubic**	***y* = 0.007*x*** ^**3 **^ **− 0.078*x*** ^**2**^ ** + 0.0686*x* + 1.8356**	**0.939**	**0.000**
20 : 5n-3	4.96 ± 0.09^a^	4.87 ± 0.12^a^	4.78 ± 0.20^a^	4.56 ± 0.23^ab^	4.04 ± 0.10^b^	**Cubic**	***y* = −0.024*x*** ^**3 **^ **+ 0.145*x*** ^**2 **^ **−** **0.3543*x* + 5.1927**	**0.689**	**0.004**
22 : 5n-3	2.25 ± 0.08	2.36 ± 0.05	2.33 ± 0.06	2.49 ± 0.08	2.57 ± 0.10	Cubic	*y* = 0.0049*x*^3^ − 0.0339*x*^2^ + 0.1322*x* + 2.1587	0.480	0.058
22 : 6n-3	18.0 ± 0.37^a^	18.4 ± 0.25^a^	17.5 ± 0.56^a^	17.1 ± 0.58^a^	14.8 ± 0.12^b^	**Cubic**	***y* = −0.0447*x*** ^**3**^ ** + 0.0558*x*** ^**2**^ ** + 0.2605*x* + 17.784**	**0.792**	**0.000**
n-3 PUFA	27.4 ± 0.48^a^	27.7 ± 0.37^a^	26.5 ± 0.58^a^	25.7 ± 0.81^a^	22.7 ± 0.29^b^	**Cubic**	***y* = −0.0591*x*** ^**3 **^ **+ 0.0911*x*** ^**2**^ ** + 0.1219*x* + 27.304**	**0.828**	**0.000**
DHA/EPA	3.63 ± 0.08	3.78 ± 0.09	3.67 ± 0.18	3.76 ± 0.16	3.67 ± 0.07	Cubic	*y* = 0.0067*x*^3^ − 0.0801*x*^2^ + 0.2842*x* + 3.4313	0.038	0.930

Data in a same row not sharing the same superscript letter were significantly different (*P*  < 0.05). TFA, total fatty acid; SFA, saturated fatty acid; MUFA, monounsaturated fatty acid; PUFA, polyunsaturated fatty acid; and LC-PUFA, long-chain polyunsaturated fatty acids. The significant regressions were marked in bold.

**Table 10:** Serum biochemical indices of juvenile tiger puffer (mean ± standard error).

Parameters	FO	25PO	50PO	75PO	100PO	Regression			
						Model	Equation	*R* ^2^	*P*
TG (mmol/L)	1.97 ± 0.23	2.05 ± 0.03	2.59 ± 0.23	2.59 ± 0.26	2.50 ± 0.22	Cubic	*y* = −0.0126*x*^3^ + 0.0392*x*^2^ + 0.303*x* + 1.4099	0.290	0.131
TC (mmol/L)	3.53 ± 0.55^b^	5.22 ± 0.41^ab^	6.66 ± 0.48^a^	4.41 ± 0.32^b^	4.58 ± 0.42^b^	**Cubic**	***y* = −0.1661*x*** ^**3**^ ** + 1.0013*x*** ^**2**^ **− 0.6386*x* + 3.4308**	**0.599**	**0.002**
TBA (mmol/L)	1.03 ± 0.00	2.26 ± 0.22	1.43 ± 0.22	1.26 ± 0.33	1.07 ± 0.27	Cubic	*y* = 0.1699*x*^3^ − 1.6662*x*^2^ + 4.7424*x* – 2.1417	0.449	0.046
MDA (mmol/L)	4.65 ± 0.32^a^	4.40 ± 0.17^a^	3.23 ± 0.35^ab^	3.31 ± 0.59^ab^	2.07 ± 0.28^b^	**Cubic**	***y* = −0.0325*x*** ^**3**^ ** + 0.2404*x*** ^**2**^ **−1.0802*x* + 5.592**	**0.627**	**0.001**
PCO (nmol/mgprot)	0.60 ± 0.13	1.07 ± 0.24	1.05 ± 0.12	0.76 ± 0.13	0.54 ± 0.11	Cubic	*y* = 0.0471*x*^3^ − 0.5425*x*^2^ + 1.7804*x* – 0.687	0.419	0.030
Glu (mmol/L)	4.67 ± 0.24	4.79 ± 0.20	4.89 ± 0.13	4.99 ± 0.08	4.84 ± 0.06	Cubic	*y* = −0.0193*x*^3^ + 0.1351*x*^2^ − 0.1696*x* + 4.7304	0.128	0.519
TP (g/L)	26.9 ± 0.93	25.9 ± 2.61	23.9 ± 1.08	25.2 ± 2.13	30.5 ± 2.03	Cubic	*y* = 0.4153*x*^3^ − 2.6187*x*^2^ + 3.7358*x* + 25.397	0.315	0.101
HDL-C (mmol/L)	2.26 ± 0.26^bc^	1.89 ± 0.21^c^	2.99 ± 0.23^ab^	3.60 ± 0.14^a^	2.84 ± 0.23^ab^	**Cubic**	***y* = −0.237*x*** ^**3**^ ** + 2.0424*x*** ^**2**^ **−4.7645*x* + 5.2024**	**0.597**	**0.000**
LDL-C (mmol/L)	1.07 ± 0.12	1.13 ± 0.14	1.38 ± 0.08	1.07 ± 0.05	0.94 ± 0.12	Cubic	*y* = 0.0026*x*^3^ − 0.0868*x*^2^ + 0.4088*x* + 0.7268	0.303	0.182

Data in a same row not sharing a same superscript letter are significantly different (*P* < 0.05). The significant regressions were marked in bold.

## Data Availability

Raw data supporting the conclusions of this manuscript will be made available by the authors, without undue reservation, to any qualified researcher.

## Supplementary Materials

Supplementary Materials The supplementary materials include Tables S1, S2, and S3, as well as Figures S1 and S2.

## References

[1] Bell J. G., Henderson R. J., Tocher D. R. (2002). Substituting fish oil with crude palm oil in the diet of Atlantic salmon (*Salmo salar*) affects muscle fatty acid composition and hepatic fatty acid metabolism. *The Journal of Nutrition*.

[2] Turchini G. M., Torstensen B. E., Ng W. K. (2009). Fish oil replacement in finfish nutrition. *Reviews in Aquaculture*.

[3] Xu H., Turchini G. M., Francis D. S. (2020). Are fish what they eat? A fatty acid’s perspective. *Progress in Lipid Research*.

[4] Oo A. N., Satoh S., Tsuchida N. (2007). Effect of replacements of fishmeal and fish oil on growth and dioxin contents of rainbow trout. *Fisheries Science*.

[5] Ng W. K., Lim P. K., Boey P. L. (2003). Dietary lipid and palm oil source affects growth, fatty acid composition and muscle alpha-tocopherol concentration of African catfish, *Clarias gariepinus*. *Aquaculture*.

[6] Rtocher D. (2003). Metabolism and functions of lipids and fatty acids in teleost fish. *Reviews in Fisheries Science*.

[7] Caballero M. J., Obach A., Rosenlund G., Montero D., Gisvold M., Izquierdo M. S. (2002). Impact of different dietary lipid sources on growth, lipid digestibility, tissue fatty acid composition and histology of rainbow trout, *Oncorhynchus mykiss*. *Aquaculture*.

[8] Tocher D. R., Zheng X., Schlechtriem C., Hastings N., Dick J. R., Teale A. J. (2006). Highly unsaturated fatty acid synthesis in marine fish: cloning, functional characterization, and nutritional regulation of fatty acyl delta 6 desaturase of Atlantic cod (*Gadus morhua* L.). *Lipids*.

[9] Majid N. A., Ramli Z., Sum S. M., Awang A. H. (2021). Sustainable palm oil certification scheme frameworks and impacts: a systematic literature review. *Sustainability*.

[10] Ng W. K., Wang Y., Ketchimenin P., Kuen K. H. (2004). Replacement of dietary fish oil with palm fatty acid distillate elevates tocopherol and tocotrienol concentrations and increases oxidative stability in the muscle of African catfish, *Clarias gariepinus*. *Aquaculture*.

[11] Ng W. K. (2002). Potential of palm oil utilisation in aquaculture feeds. *Asia Pacific Journal of Clinical Nutrition*.

[12] Turchini G. M., Francis D. S., Senadheera S. P. S. D., Thanuthong T., De Silva S. S. (2011). Fish oil replacement with different vegetable oils in Murray cod: evidence of an “omega-3 sparing effect, by other dietary fatty acids. *Aquaculture*.

[13] Tocher D., Betancor M., Sprague M., Olsen R. E., Napier J. A. (2019). Omega-3 long-chain polyunsaturated fatty acids, EPA and DHA: bridging the gap between supply and demand. *Nutrients*.

[14] Tocher D. R. (2015). Omega-3 long-chain polyunsaturated fatty acids and aquaculture in perspective. *Aquaculture*.

[15] Turchini G. M., Francis D. S., Silva S. D. (2006). Modification of tissue fatty acid composition in Murray cod (*Maccullochella peelii peelii*, Mitchell) resulting from a shift from vegetable oil diets to a fish oil diet. *Aquaculture Research*.

[16] Turchini G. M., Moretti V. M., Hermon K. (2013). Monola oil versus canola oil as a fish oil replacer in rainbow trout feeds: effects on growth, fatty acid metabolism and final eating quality. *Food Chemistry*.

[17] Whelan J. (2008). The health implications of changing linoleic acid intakes. *Prostagkandins Leukotrienes and Essential Fatty Acids*.

[18] Ramsden C. E., Hibbelin J. R., Lands W. E. (2009). Letter to the editor re: linoleic acid and coronary heart disease. *Prostagkandins Leukotrienes and Essential Fatty Acids*.

[19] Fountoulaki E., Vasilaki A., Hurtado R. (2009). Fish oil substitution by vegetable oils in commercial diets for gilthead sea bream (*Sparus aurata* L.); effects on growth performance, flesh quality and fillet fatty acid profile recovery of fatty acid profiles by a fish oil finishing diet under fluctuating water temperatures. *Aquaculture*.

[20] Ooi Z. X., Teoh Y. P., Kunasundari B., Shuit S. H. (2017). Oil palm frond as a sustainable and promising biomass source in Malaysia: a review. *Environmental Progress & Sustainable Energy*.

[21] Madrigal J. F., Kaealazos V., Campbell P. J., Bell J. G., Tocher D. R. (2005). Influence of dietary palm oil on growth, tissue fatty acid compositions, and fatty acid metabolism in liver and intestine in rainbow trout (*Oncorhynchus mykiss*). *Aquaculture Nutrition*.

[22] Gao J., Koshio S., Ishikawa M., Ren T., Komilus C. F., Han Y. (2012). Effects of dietary palm oil supplements with oxidized and non-oxidized fish oil on growth performances and fatty acid compositions of juvenile Japanese sea bass, *Lateolabrax japonicus*. *Aquaculture*.

[23] Pettersson A., Pickova J., Brannas E. (2010). Swimming performance at different temperatures and fatty acid composition of Arctic charr (*Salvelinus alpinus*) fed palm and rapeseed oils. *Aquaculture*.

[24] Monge-ortiz R., Tomas-Vidal A., Rodriguez-Barreto D. (2018). Replacement of fish oil with vegetable oil blends in feeds for greater amberjack (*Seriola dumerili*) juveniles: effect on growth performance, feed efficiency, tissue fatty acid composition and flesh nutritional value. *Aquaculture Nutrition*.

[25] Asdari R., Aliyu-Paiko M., Hashim R. (2011). Effects of different dietary lipid sources in the diet for *Pangasius nasutus* (Bleeker, 1863) juveniles on growth performance, feed efficiency, body indices and muscle and liver fatty acid compositions. *Aquaculture Nutrition*.

[26] Aliyu-Paiko M., Hashim R. (2012). Effects of substituting dietary fish oil with crude palm oil and palm fatty acid distillate on growth, muscle fatty acid composition and the activities of hepatic lipogenic enzymes in snakehead (*Channa striatus*, Bloch 1793) fingerling. *Aquaculture Research*.

[27] Safiin N. S. Z., Ching F. F., Shapawi R. (2022). Successful co-feeding of Asian Seabass, *Lates calcarifer* larvae with palm oil-based microdiets and live feeds. *Frontiers in Sustainable Food Systems*.

[28] Rostika R., Safitri R. (2012). Influence of fish feed containing corn-cob was fermented by *Trichoderma Sp*, *Aspergillus Sp*, *Rhizopus oligosporus* to the rate of growth of Java barb (*Puntius gonionitus*). *Apcbee Procedia*.

[29] Han Y. Z., Jiang Z. Q., Ren T. J. (2015). Effect of dietary fish oil replacement with palm oil on growth performance, hematology and liver anti-oxidative enzymes of juvenile Japanese flounder *Paralichthys olivaceus* (Temminck & Schlegel, 1846). *Journal of Appled Ichthyology*.

[30] Huang Y., Wen X., Li S., Li W. (2016). Effects of dietary fish oil replacement with palm oil on the growth, feed utilization, biochemical composition, and antioxidant status of juvenile Chu’s Croaker, *Nibea coibor*. *Journal of the World Aquaculture Society*.

[31] Mu H., Wei C., Zhang Y. (2020). Impacts of replacement of dietary fish oil by vegetable oils on growth performance, anti-oxidative capacity, and inflammatory response in large yellow croaker *Larimichthys crocea*. *Fish Physiology and Biochemistry*.

[32] Kikuchi K., Furuta T., Iwata N., Onuki K., Noguchi T., Sugita H. (2011). Effect of dietary fatty acid composition on the growth of the tiger puffer *Takifugu rubripes*. *Fisheries Sciences*.

[33] Kikuchi K., Furuta T., Iwata N., Onuki K., Noguchi T. (2009). Effect of dietary lipid levels on the growth, feed utilization, body composition and blood characteristics of tiger puffer *Takifugu rubripes*. *Aquaculture*.

[34] Miyajima-Taga Y., Masuda R., Kurihara A., Komi R., Yamashita Y., Takeuchi T. (2018). Efficacy of feeding tiger puffer *Takifugu rubripes* on moon jellyfish with respect to nutritional composition and behavioural traits. *Aquaculture Nutrition*.

[35] Lu J., Zheng J., Liu H., Li J., Chen H., Chen K. (2010). Protein profiling analysis of skeletal muscle of a pufferfish, *Takifugu rubripes*. *Molecular Biology Reports*.

[36] Zhang F., Li L., Meng X. (2023). Feeding strategy to use beef tallow and modify farmed tiger puffer fatty acid composition. *Animals*.

[37] Li L., Zhang F., Meng X. (2022). Fish oil replacement with poultry oil in the diet of tiger puffer (*Takifugu rubripes*): effects on growth performance, body composition, and lipid metabolism. *Aquaculture Nutrition*.

[38] Liu G., Li L., Song S. (2024). Marine fish oil replacement with lard or basa fish (*Pangasius bocourti*) offal oil in the diet of tiger puffer (*Takifugu rubripes*): effects on growth performance, body composition, and flesh quality. *Animals*.

[39] Li L., Zhang F., Meng X. (2023). Recovery of fatty acid and volatile flavor compound composition in farmed tiger puffer (*Takifugu rubripes*) with a fish oil-finishing strategy. *Marine Drugs*.

[40] Zhao L., Li L., Zhang F. (2024). Combined replacement of fishmeal and fish oil by poultry byproduct meal and mixed oil: effects on the growth performance, body composition, and muscle quality of tiger puffer. *Aquaculture Nutrition*.

[41] Xu H., Zhang Y., Zhang Y. (2016). Graded levels of fish protein hydrolysate in high plant diets for turbot (*Scophthalmus maximus*): effects on growth performance and lipid accumulation. *Aquaculture*.

[42] Liao Z., Sun Z., Bi Q. (2021). Screening of reference genes in tiger puffer (*Takifugu rubripes*) across tissues and under different nutritional conditions. *Fish Physiology and Biochemistry*.

[43] Miller M. R., Nichols P. D., Carter C. G. (2008). n-3 Oil sources for use in aquaculture—alternatives to the unsustainable harvest of wild fish. *Nutrition Research Reviews*.

[44] Tocher D. R. (2010). Fatty acid requirements in ontogeny of marine and freshwater fish. *Aquaculture Research*.

[45] Turchini G. M., Moretti V. M., Mentasti T., Orban E., Valfre F. (2007). Effects of dietary lipid source on fillet chemical composition, flavour volatile compounds and sensory characteristics in the freshwater fish tench (*Tinca tinca* L.). *Food Chemistry*.

[46] Kaneko G., Yamada T., Han Y. (2013). Differences in lipid distribution and expression of peroxisome proliferator-activated receptor gamma and lipoprotein lipase genes in Torafugu and red seabream. *General and Comparative Endocrinology*.

[47] Karalazos V., Treasurer J., Cutts C. J. (2007). Effects of fish meal replacement with full-fat soy meal on growth and tissue fatty acid composition in Atlantic cod (*Gadus morhua*). *Journal of Agriculture and Food Chemistry*.

[48] Morais S., Bell J. G., Robertson D. A., Roy W. J., Morris P. C. (2001). Protein/lipid ratios in extruded diets for Atlantic cod (*Gadus morhua* L.): effects on growth, feed utilisation, muscle composition and liver histology. *Aquaculture*.

[49] Xu H., Meng X., Jia L., Wei Y., Sun B., Liang M. (2020). Tissue distribution of transcription for 29 lipid metabolism-related genes in *Takifugu rubripes*, a marine teleost storing lipid predominantly in liver. *Fish Physiology and Biochemistry*.

[50] Tiku P. E., Gracey A. Y., Macartney A. I., Beynon R. J., Cossins A. R. (1996). Cold-induced expression of delta(9)-desaturase in carp by transcriptional and posttranslational mechanisms. *Science*.

[51] Stubhaug I., Lie Ø., Torstensen B. E. (2007). Fatty acid productive value and *β*-oxidation capacity in Atlantic salmon (*Salmo salar* L.) fed on different lipid sources along the whole growth period. *Aquaculture Nutrition*.

[52] Liu Y., Wen J. J., Ning L. J. (2019). Comparison of effects of dietary-specific fatty acids on growth and lipid metabolism in Nile tilapia. *Aquaculture Nutrition*.

[53] Song Y., Wang L. J., Luo Z., Hogstrand C., Lai X., Zheng F. (2024). Moderate replacement of fish oil with palmitic acid-stimulated mitochondrial fusion promotes *β*-oxidation by Mfn2 interacting with Cpt1*α* via its GTPase-domain. *The Journal of Nutritional Biochemistry*.

[54] Song Z., Xiong H., Meng X. (2023). Dietary cholesterol supplementation inhibits the steroid biosynthesis but does not affect the cholesterol transport in two marine teleosts: a hepatic transcriptome study. *Aquaculture Nutrition*.

[55] Xue B., Lv H., Liu Y., Gao Y. (2022). Effects of terrestrial lipid blend on the growth performance, body composition, nonspecific immunity, antioxidant status and stress resistance when the dietary n-3 long-chain polyunsaturated fatty acids requirements are met in juvenile Japanese sea bass (*Lateolabrax japonicus*). *Aquaculture Research*.

