# Supplementary material for: Efficacy of Palm Oil Application in Tiger Puffer Diets: Growth, Body Composition, Muscle Texture, and Lipid Metabolism
Source: Aquac Nutr. 2024 Aug 12;2024:2709579. doi: 10.1155/2024/2709579 (PMC11333123; doi:10.1155/2024/2709579)
Supplement: Supplementary Materials — The supplementary materials include Tables S1, S2, and S3, as well as Figures S1 and S2. [file 2709579.f1.docx]

Supplementary Table S1 Relative mRNA expression of lipid metabolism genes in the liver of tiger puffer.

| Parameters | FO | 25PO | 50PO | 75PO | 100PO | Regression | | | |  |
| --- | --- | --- | --- | --- | --- | --- | --- | --- | --- | --- |
|  |  |  |  |  |  | Model | Equation | R^2^ | P |  |
| Lipogenesis | | | | | | | | | | |
| *acacβ* | 1.00±0.07 | 1.11±0.07 | 1.19±0.19 | 1.27±0.12 | 0.82±0.16 | cubic | y = -0.042x^3^ + 0.2953x^2^ - 0.5159x + 1.2681 | 0.268 | 0.071 |  |
| *fas* | 1.00±0.13^b^ | 1.01±0.15^b^ | 1.36±0.32^ab^ | 2.14±0.29^a^ | 0.97±0.23^b^ | **cubic** | **y = -0.1908x^3^ + 1.5791x^2^ - 3.5676x + 3.215** | **0.499** | **0.010** |  |
| *scd* | 1.00±0.16^a^ | 0.66±0.16^ab^ | 0.71±0.14^ab^ | 0.78±0.16^ab^ | 0.34±0.10^b^ | **cubic** | **y = -0.0746x^3^ + 0.6594x^2^ - 1.8076x + 2.2248** | **0.334** | **0.019** |  |
| β-oxidation | | | | | | | | | | |
| *cpt-1* | 1.00±0.15^b^ | 1.24±0.17^ab^ | 1.86±0.33^ab^ | 2.12±0.05^a^ | 1.46±0.27^ab^ | **cubic** | **y = -0.109x^3^ + 0.8265x^2^ - 1.466x + 1.7445** | **0.512** | **0.003** |  |
| *acox1* | 1.00±0.10^ab^ | 1.36±0.11^a^ | 1.33±0.16^a^ | 1.31±0.12^a^ | 0.60±0.10^b^ | **cubic** | **y = -0.0222x^3^ + 0.0489x^2^ + 0.2965x + 0.6907** | **0.521** | **≤ 0.001** |  |
| *acox3* | 1.00±0.29 | 1.12±0.31 | 1.10±0.32 | 1.71±0.26 | 0.94±0.34 | cubic | y = -0.1033x^3^ + 0.848x^2^ - 1.9004x + 2.1967 | 0.101 | 0.423 |  |
| *acaa1* | 1.00±0.05 | 1.16±0.19 | 1.33±0.17 | 1.60±0.29 | 1.03±0.22 | cubic | y = -0.0714x^3^ + 0.5467x^2^ - 1.0589x + 1.6032 | 0.208 | 0.208 |  |
| *acaa2* | 1.00±0.22^b^ | 1.84±0.45^ab^ | 1.52±0.26^ab^ | 2.01±0.32^ab^ | 2.89±0.63^a^ | **cubic** | **y = 0.0672x^3^ - 0.532x^2^ + 1.5802x - 0.1099** | **0.438** | **0.011** |  |
| Biosynthesis of glycerides | | | | | | | | | | |
| *dgat1* | 1.00±0.18^ab^ | 1.04±0.07^ab^ | 1.25±0.26^a^ | 0.88±0.21^ab^ | 0.46±0.11^b^ | **cubic** | **y = -0.0181x^3^ + 0.0589x^2^ + 0.0753x + 0.8617** | **0.441** | **0.004** |  |
| *mgat* | 1.00±0.15 | 0.68±0.08 | 0.73±0.15 | 1.21±0.20 | 0.76±0.29 | cubic | y = -0.1088x^3^ + 0.9929x^2^ - 2.6457x + 2.7916 | 0.194 | 0.167 |  |
| Hydrolysis of glycerides | | | | | | | | | | |
| *mgll* | 1.00±0.13 | 0.93±0.18 | 1.05±0.17 | 0.87±0.15 | 1.21±0.13 | cubic | y = 0.0274x^3^ - 0.2091x^2^ + 0.4585x + 0.7047 | 0.067 | 0.608 |  |
| *hsl* | 1.00±0.09 | 1.40±0.15 | 1.29±0.12 | 1.02±0.14 | 1.52±0.12 | cubic | y = 0.1076x^3^ - 0.9654x^2^ + 2.5877x - 0.7429 | 0.335 | 0.019 |  |
| *atgl* | 1.00±0.34 | 1.90±0.26 | 1.65±0.50 | 1.49±0.54 | 0.99±0.46 | cubic | y = 0.0686x^3^ - 0.8174x^2^ + 2.7745x - 1.0059 | 0.146 | 0.313 |  |
| *daglα* | 1.00±0.13 | 0.92±0.15 | 0.79±0.09 | 0.81±0.03 | 0.79±0.12 | cubic | y = 0.0012x^3^ + 0.0083x^2^ - 0.1413x + 1.139 | 0.094 | 0.454 |  |
| Lipid digestion | | | | | | | | | | |
| *bsal* | 1.00±0.29 | 1.06±0.28 | 0.72±0.23 | 0.72±0.16 | 0.63±0.16 | cubic | y = 0.0246x^3^ - 0.2191x^2^ + 0.4613x + 0.7477 | 0.083 | 0.512 |  |
| *pl* | 1.00±0.24 | 1.08±0.23 | 1.13±0.28 | 0.76±0.13 | 0.79±0.05 | cubic | y = 0.0315x^3^ - 0.323x^2^ + 0.9072x + 0.3654 | 0.084 | 0.615 |  |
| Lipid transportation | | | | | | | | | | |
| *apoa1* | 1.00±0.10^b^ | 1.46±0.30^ab^ | 1.64±0.15^ab^ | 2.45±0.39^a^ | 1.62±0.33^ab^ | **cubic** | **y = -0.1323x^3^ + 1.0554x^2^ - 2.064x + 2.2181** | **0.290** | **0.038** |  |
| *lpl* | 1.00±0.05^ab^ | 1.19±0.21^ab^ | 1.26±0.17^a^ | 1.05±0.18^ab^ | 0.56±0.07^b^ | **cubic** | **y = -0.0129x^3^ - 0.0023x^2^ + 0.3013x + 0.7119** | **0.447** | **0.007** |  |
| *apob100* | 1.00±0.09 | 0.91±0.10 | 0.98±0.11 | 0.76±0.08 | 0.75±0.07 | cubic | y = 0.0042x^3^ - 0.0464x^2^ + 0.0877x + 0.939 | 0.165 | 0.189 |  |
| *apoe1* | 1.00±0.19^b^ | 1.33±0.25^ab^ | 1.02±0.12^ab^ | 1.22±0.22^ab^ | 2.14±0.38^a^ | **cubic** | **y = 0.1131x^3^ - 0.901x^2^ + 2.1835x - 0.3824** | **0.359** | **0.015** |  |
| *lipc* | 1.00±0.19^b^ | 1.37±0.22^ab^ | 1.90±0.13^ab^ | 2.05±0.34^a^ | 2.08±0.22^a^ | **cubic** | **y = -0.0264x^3^ + 0.1603x^2^ + 0.1288x + 0.7251** | **0.449** | **0.006** |  |
| *mttp* | 1.00±0.15^a^ | 1.40±0.26^a^ | 1.43±0.07^a^ | 2.16±0.15^b^ | 0.91±0.13^a^ | **cubic** | **y = -0.134x^3^ + 1.0214x^2^ - 1.9963x + 2.1623** | **0.467** | **0.001** |  |
| *fabp10a* | 1.00±0.23 | 1.07±0.36 | 0.91±0.21 | 1.76±0.52 | 2.00±0.49 | cubic | y = -0.0421x^3^ + 0.4886x^2^ - 1.3786x + 1.9678 | 0.265 | 0.111 |  |
| Lipid metabolism-related transcriptional factors | | | | | | | | | | |
| *srebf1* | 1.00±0.12 | 0.78±0.08 | 0.85±0.06 | 0.87±0.04 | 0.75±0.05 | cubic | y = -0.0363x^3^ + 0.3361x^2^ - 0.9544x + 1.6493 | 0.272 | 0.136 |  |
| *pparα1* | 1.00±0.25 | 0.90±0.13 | 1.05±0.16 | 1.18±0.19 | 0.88±0.19 | cubic | y = -0.0562x^3^ + 0.4762x^2^ - 1.1454x + 1.7305 | 0.081 | 0.611 |  |
| *pparα2* | 1.00±0.09 | 1.02±0.26 | 1.08±0.33 | 1.08±0.18 | 0.75±0.09 | cubic | y = -0.0328x^3^ + 0.2402x^2^ - 0.489x + 1.2861 | 0.079 | 0.588 |  |
| *fxr* | 1.00±0.11 | 2.01±0.50 | 3.50±0.55 | 3.55±1.04 | 1.16±0.41 | cubic | y = -0.2426x^3^ + 1.5928x^2^ - 1.995x + 1.6295 | 0.602 | 0.006 |  |
| *lrh-1* | 1.00±0.11^b^ | 1.39±0.15^ab^ | 1.28±0.16^ab^ | 2.04±0.33^a^ | 1.18±0.26^ab^ | **cubic** | **y = -0.0936x^3^ + 0.7254x^2^ - 1.4044x + 1.8257** | **0.231** | **0.073** |  |
| *hnf4α* | 1.00±0.20 | 1.17±0.18 | 1.30±0.17 | 1.62±0.22 | 0.96±0.28 | cubic | y = -0.0781x^3^ + 0.5987x^2^ - 1.1821x + 1.6848 | 0.183 | 0.160 |  |
| Cholesterol biosynthesis | | | | | | | | | | |
| *hmgcr* | 1.00±0.17^b^ | 0.99±0.3^b^ | 0.98±0.48^b^ | 4.18±0.83^a^ | 1.05±0.28^b^ | **cubic** | **y = -0.589x^3^ + 5.0758x^2^ - 12.208x + 9.019** | **0.399** | **0.006** |  |
| *dhcr24* | 1.00±0.09^b^ | 1.25±0.16^b^ | 1.30±0.37^b^ | 2.94±0.56^a^ | 1.29±0.17^b^ | **cubic** | **y = -0.2568x^3^ + 2.1668x^2^ - 4.9687x + 4.2021** | **0.251** | **0.021** |  |
| *lss* | 1.00±0.15^b^ | 2.24±0.63^b^ | 1.88±0.30^b^ | 5.28±1.11^a^ | 2.81±0.87^ab^ | **cubic** | **y = -0.2593x^3^ + 2.1638x^2^ - 4.4722x + 3.8128** | **0.346** | **0.006** |  |
| *dhcr7* | 1.00±0.10 | 1.99±0.42 | 1.32±0.25 | 5.12±1.16 | 2.67±0.80 | cubic | y = -0.3963x^3^ + 3.3233x^2^ - 7.2838x + 5.5727 | 0.325 | 0.015 |  |
| *sc5d* | 1.00±0.12^c^ | 2.24±0.45^bc^ | 2.38±0.43^b^ | 3.95±0.52^a^ | 0.95±0.20^c^ | **cubic** | **y = -0.2635x^3^ + 1.8686x^2^ - 3.056x + 2.5254** | **0.632** | **≤ 0.001** |  |
| *msmo* | 1.00±0.23^b^ | 1.33±0.42^ab^ | 1.98±0.65^ab^ | 3.79±1.01^a^ | 2.81±0.68^ab^ | **cubic** | **y = -0.2368x^3^ + 2.0364x^2^ - 4.4227x + 3.6823** | **0.347** | **0.018** |  |
| Cholesterol transport-related | | | | | | | | | | |
| *acat1* | 1.00±0.14 | 1.52±0.17 | 1.03±0.19 | 1.44±0.24 | 1.00±0.14 | cubic | y = 0.0146x^3^ - 0.2048x^2^ + 0.7773x + 0.4637 | 0.074 | 0.567 |  |
| *acat2* | 1.00±0.06 | 0.97±0.14 | 1.18±0.21 | 1.59±0.35 | 1.14±0.35 | cubic | y = -0.1063x^3^ + 0.8893x^2^ - 2.0458x + 2.2831 | 0.148 | 0.253 |  |

Data in a same row not sharing a same superscript letter are significantly different (*P*˂0.05). The significant regressions were marked in bold.

Supplementary Table S2 Relative mRNA expression of lipid metabolism genes in the muscle of tiger puffer (mean ± standard error)

| Parameters | FO | 25PO | 50PO | 75PO | 100PO | Regression | | | |
| --- | --- | --- | --- | --- | --- | --- | --- | --- | --- |
|  |  |  |  |  |  | Model | Equation | R^2^ | P |
| Lipogenesis | | | | | | | | | |
| *acacβ* | 1.00±0.13 | 1.20±0.16 | 1.40±0.16 | 1.02±0.07 | 1.52±0.18 | cubic | y = 0.0738x^3^ - 0.6622x^2^ + 1.8174x - 0.2577 | 0.207 | 0.106 |
| *fas* | 1.00±0.08^b^ | 1.20±0.09^b^ | 1.37±0.09^ab^ | 1.19±0.10^b^ | 1.76±0.22^a^ | **cubic** | **y = 0.0646x^3^ - 0.5537x^2^ + 1.51x - 0.0427** | **0.398** | **0.004** |
| *scd* | 1.00±0.07 | 1.07±0.18 | 0.95±0.12 | 1.05±0.18 | 0.92±0.17 | cubic | y = -0.0031x^3^ + 0.0136x^2^ - 0.006x + 1.0081 | 0.012 | 0.964 |
| β-oxidation | | | | | | | | | |
| *cpt-1* | 1.00±0.08 | 1.28±0.20 | 1.36±0.11 | 1.08±0.12 | 1.18±0.17 | cubic | y = 0.0493x^3^ - 0.4949x^2^ + 1.4874x - 0.056 | 0.120 | 0.336 |
| *acox1* | 1.00±0.10 | 1.21±0.09 | 0.91±0.08 | 0.92±0.09 | 1.09±0.05 | cubic | y = 0.0557x^3^ - 0.4836x^2^ + 1.199x + 0.2413 | 0.213 | 0.096 |
| *acox3* | 1.00±0.13^ab^ | 0.83±0.13^ab^ | 0.70±0.12^b^ | 0.75±0.04^ab^ | 1.20±0.13^a^ | **cubic** | **y = 0.0296x^3^ - 0.1651x^2^ + 0.1237x + 1.012** | **0.339** | **0.012** |
| *acaa1* | 1.00±0.11^b^ | 1.12±0.12^b^ | 1.27±0.11^b^ | 0.88±0.06^b^ | 1.72±0.07^a^ | **cubic** | **y = 0.1006x^3^ - 0.839x^2^ + 2.0971x - 0.39** | **0.527** | **≤ 0.001** |
| *acaa2* | 1.00±0.09^ab^ | 1.11±0.10^a^ | 0.97±0.04^ab^ | 0.99±0.07^ab^ | 0.72±0.03^b^ | **cubic** | **y = -0.0023x^3^ - 0.0215x^2^ + 0.1288x + 0.9079** | **0.365** | **0.009** |
| Biosynthesis of glycerides | | | | | | | | | |
| *dgat1* | 1.00±0.06 | 1.05±0.15 | 0.80±0.08 | 0.89±0.09 | 1.09±0.07 | cubic | y = 0.0342x^3^ - 0.2609x^2^ + 0.5266x + 0.715 | 0.167 | 0.199 |
| *mgat* | 1.00±0.11 | 0.73±0.06 | 0.64±0.05 | 0.64±0.07 | 1.00±0.14 | cubic | y = 0.0155x^3^ - 0.0452x^2^ - 0.2104x + 1.2321 | 0.384 | 0.006 |
| Hydrolysis of glycerides | | | | | | | | | |
| *mgll* | 1.00±0.10 | 0.87±0.09 | 1.02±0.13 | 0.84±0.09 | 1.24±0.15 | cubic | y = 0.0269x^3^ - 0.1899x^2^ + 0.3679x + 0.7706 | 0.162 | 0.221 |
| *hsl* | 1.00±0.09 | 1.07±0.11 | 1.07±0.08 | 1.02±0.08 | 1.37±0.15 | cubic | y = 0.0407x^3^ - 0.3292x^2^ + 0.8084x + 0.4727 | 0.257 | 0.056 |
| *atgl* | 1.00±0.11 | 1.55±0.38 | 1.52±0.24 | 1.43±0.31 | 1.90±0.25 | cubic | y = 0.1185x^3^ - 1.072x^2^ + 2.9846x - 1.041 | 0.198 | 0.120 |
| *daglα* | 1.00±0.09^b^ | 1.03±0.13^b^ | 1.03±0.09^b^ | 1.03±0.09^b^ | 1.66±0.22^a^ | cubic | **y = 0.033x^3^ - 0.2526x^2^ + 0.5859x + 0.6265** | **0.179** | **0.170** |
| Lipid digestion | | | | | | | | | |
| *bsal* | 1.00±0.29 | 0.76±0.18 | 0.78±0.15 | 1.12±0.37 | 0.86±0.16 | cubic | y = -0.0677x^3^ + 0.6307x^2^ - 1.7201x + 2.1746 | 0.068 | 0.681 |
| *pl* | 1.00±0.07^ab^ | 0.66±0.06^b^ | 0.74±0.13^b^ | 0.77±0.01^b^ | 1.20±0.09^a^ | **cubic** | **y = -0.0016x^3^ + 0.1183x^2^ - 0.6116x + 1.4859** | **0.589** | **0.005** |
| Lipid transportation | | | | | | | | | |
| *lpl* | 1.00±0.16 | 0.44±0.22 | 1.32±0.48 | 0.90±0.36 | 1.04±0.23 | cubic | y = -0.0525x^3^ + 0.4977x^2^ - 1.3472x + 1.8582 | 0.033 | 0.893 |
| *apob100* | 1.00±0.12 | 1.30±0.34 | 1.37±0.27 | 0.95±0.12 | 1.64±0.22 | cubic | y = 0.1107x^3^ - 0.9659x^2^ + 2.5249x - 0.6912 | 0.195 | 0.151 |
| *mttp* | 1.11±0.06^ab^ | 0.74±0.06^b^ | 0.93±0.13^b^ | 0.97±0.08^b^ | 1.42±0.16^a^ | **cubic** | **y = -0.0132x^3^ + 0.1978x^2^ - 0.7118x + 1.4977** | **0.402** | **0.011** |
| *fabp1* | 1.00±0.12^b^ | 1.50±0.12^ab^ | 1.09±0.12^b^ | 1.42±0.15^ab^ | 1.95±0.18^a^ | **cubic** | **y = 0.0921x^3^ - 0.7619x^2^ + 1.9514x - 0.2431** | **0.544** | **0.002** |
| *fatp1* | 1.00±0.04 | 1.09±0.05 | 0.81±0.09 | 0.95±0.10 | 0.97±0.12 | cubic | y = 0.0154x^3^ - 0.1263x^2^ + 0.2583x + 0.8727 | 0.067 | 0.609 |
| Lipid metabolism-related transcriptional factors | | | | | | | | | |
| *srebf1* | 1.00±0.04^b^ | 1.05±0.10^ab^ | 0.94±0.11^b^ | 0.97±0.06^b^ | 1.46±0.20^a^ | **cubic** | **y = 0.0565x^3^ - 0.4317x^2^ + 0.9607x + 0.4162** | **0.387** | **0.006** |
| *pparα1* | 1.00±0.07 | 0.75±0.07 | 0.73±0.05 | 0.70±0.08 | 0.93±0.12 | cubic | y = 0.0022x^3^ + 0.0467x^2^ - 0.3667x + 1.3096 | 0.263 | 0.051 |
| *pparα2* | 1.00±0.12 | 1.18±0.18 | 1.24±0.19 | 1.04±0.21 | 1.45±0.09 | cubic | y = 0.0600x^3^ - 0.5271x^2^ + 1.4154x + 0.0331 | 0.137 | 0.288 |
| Cholesterol biosynthesis | | | | | | | | | |
| *lss* | 1.00±0.08^b^ | 1.08±0.16^b^ | 0.73±0.10^b^ | 1.01±0.11^b^ | 2.25±0.26^a^ | **cubic** | **y = 0.0908x^3^ - 0.6481x^2^ + 1.3111x + 0.2607** | **0.517** | **≤ 0.001** |
| *sc5d* | 1.00±0.06 | 1.13±0.14 | 0.88±0.09 | 0.89±0.10 | 0.97±0.07 | cubic | y = 0.0379x^3^ - 0.3296x^2^ + 0.7942x + 0.5103 | 0.120 | 0.336 |
| *msmo* | 1.00±0.15 | 1.32±0.31 | 1.33±0.17 | 1.49±0.14 | 1.00±0.14 | cubic | y = -0.0292x^3^ + 0.1584x^2^ - 0.0478x + 0.9417 | 0.151 | 0.244 |
| Cholesterol transport-related | | | | | | | | | |
| *acat1* | 1.00±0.06^ab^ | 1.12±0.16^ab^ | 1.14±0.16^ab^ | 0.87±0.09^b^ | 1.64±0.25^a^ | **cubic** | **y = 0.0957x^3^ - 0.7903x^2^ + 1.9374x - 0.2704** | **0.317** | **0.021** |
| *acat2* | 1.00±0.08 | 0.79±0.06 | 0.88±0.04 | 0.78±0.13 | 1.15±0.11 | cubic | y = 0.0151x^3^ - 0.0677x^2^ - 0.0262x + 1.0623 | 0.260 | 0.046 |
| *lcat* | 1.00±0.11 | 1.09±0.11 | 0.97±0.07 | 1.02±0.12 | 0.84±0.09 | cubic | y = -0.0019x^3^ - 0.0089x^2^ + 0.0747x + 0.9477 | 0.083 | 0.513 |

Data in a same row not sharing a same superscript letter are significantly different (*P*˂0.05). The significant regressions were marked in bold.

Supplementary Table S3 Relative mRNA expression of lipid metabolism genes in the intestine of tiger puffer (mean ± standard error)

| Parameters | FO | 25PO | 50PO | 75PO | 100PO | Regression | | | |
| --- | --- | --- | --- | --- | --- | --- | --- | --- | --- |
|  |  |  |  |  |  | Model | Equation | R^2^ | P |
| Lipogenesis | | | | | | | | | |
| *acacβ* | 1.00±0.07^a^ | 1.08±0.05^a^ | 0.74±0.04^b^ | 0.68±0.02^b^ | 0.65±0.06^b^ | **cubic** | **y = 0.0409x^3^ - 0.3539x^2^ + 0.7818x + 0.5500** | **0.551** | **≤ 0.001** |
| *fas* | 1.00±0.10 | 0.99±0.07 | 0.61±0.11 | 0.74±0.08 | 0.98±0.11 | cubic | y = 0.0392x^3^ - 0.2793x^2^ + 0.4549x + 0.8067 | 0.286 | 0.030 |
| *scd* | 1.00±0.09^b^ | 2.17±0.03^a^ | 1.36±0.15^b^ | 1.42±0.16^b^ | 1.11±0.07^b^ | **cubic** | **y = 0.1717x^3^ - 1.7007x^2^ + 4.8758x - 2.1809** | **0.383** | **0.010** |
| β-oxidation | | | | | | | | | |
| *cpt-1* | 1.00±0.09 | 1.06±0.07 | 0.96±0.06 | 1.08±0.05 | 0.94±0.11 | cubic | y = -0.0073x^3^ + 0.0596x^2^ - 0.1311x + 1.0961 | 0.004 | 0.993 |
| *acox1* | 1.00±0.13^a^ | 1.07±0.09^a^ | 0.38±0.05^b^ | 0.36±0.02^b^ | 0.36±0.05^b^ | **cubic** | **y = 0.0567x^3^ - 0.474x^2^ + 0.9203x + 0.5425** | **0.696** | **≤ 0.001** |
| *acox3* | 1.00±0.07^a^ | 0.91±0.04^ab^ | 0.81±0.07^ab^ | 0.68±0.01^b^ | 0.79±0.06^ab^ | **cubic** | **y = 0.0213x^3^ - 0.1634x^2^ + 0.2679x + 0.8725** | **0.561** | **0.004** |
| *acaa1* | 1.00±0.09^a^ | 1.04±0.07^a^ | 0.30±0.04^b^ | 0.27±0.05^b^ | 0.30±0.03^b^ | **cubic** | **y = 0.0686x^3^ - 0.566x^2^ + 1.0907x + 0.4528** | **0.801** | **≤ 0.001** |
| *acaa2* | 1.00±0.07^a^ | 1.69±0.09^a^ | 13.5±0.83^b^ | 14.4±1.10^b^ | 10.9±1.52^b^ | **cubic** | **y = -1.2684x^3^ + 9.9966x^2^ - 18.073x + 9.5386** | **0.796** | **≤ 0.001** |
| *ehhadh* | 1.00±0.06^c^ | 1.33±0.11^c^ | 2.04±0.10^ab^ | 2.16±0.21^a^ | 1.52±0.14^bc^ | **cubic** | **y = -0.1053x^3^ + 0.766x^2^ - 1.2088x + 1.543** | **0.610** | **≤ 0.001** |
| Biosynthesis of glycerides | | | | | | | | | |
| *dgat1* | 1.00±0.07 | 1.04±0.08 | 1.26±0.14 | 1.28±0.18 | 1.22±0.09 | cubic | y = -0.0217x^3^ + 0.1664x^2^ - 0.2719x + 1.1217 | 0.154 | 0.219 |
| *mgat2a* | 1.00±0.06^a^ | 0.84±0.07^ab^ | 0.89±0.09^a^ | 0.74±0.05^ab^ | 0.57±0.06^b^ | **cubic** | **y = -0.0185x^3^ + 0.1511x^2^ - 0.4393x + 1.2956** | **0.567** | **0.005** |
| Hydrolysis of glycerides | | | | | | | | | |
| *mgll* | 1.00±0.08^a^ | 0.78±0.09^b^ | 0.41±0.04^c^ | 0.40±0.03^c^ | 0.50±0.04^c^ | **cubic** | **y = 0.0135x^3^ - 0.0462x^2^ - 0.2655x + 1.3105** | **0.748** | **≤ 0.001** |
| *hsl* | 1.00±0.11^a^ | 0.90±0.08^a^ | 0.58±0.03^b^ | 0.57±0.04^b^ | 0.58±0.05^b^ | **cubic** | **y = 0.0188x^3^ - 0.1319x^2^ + 0.101x + 1.0295** | **0.598** | **≤ 0.001** |
| *atgl* | 1.00±0.05^a^ | 0.54±0.05^b^ | 0.47±0.07^b^ | 0.36±0.06^b^ | 0.52±0.06^b^ | **cubic** | **y = -0.0142x^3^ + 0.1998x^2^ - 0.8961x + 1.6931** | **0.676** | **≤ 0.001** |
| *daglα* | 1.00±0.05 | 1.24±0.10 | 0.52±0.05 | 1.43±0.04 | 0.92±0.06 | cubic | y = -0.0239x^3^ + 0.2176x^2^ - 0.5863x + 1.4783 | 0.017 | 0.975 |
| Lipid transportation | | | | | | | | | |
| *apoa1* | 1.00±0.10^c^ | 1.37±0.27^bc^ | 3.48±0.24^a^ | 2.99±0.65^ab^ | 2.93±0.34^ab^ | **cubic** | **y = -0.1009x^3^ + 0.6458x^2^ - 0.238x + 0.4854** | **0.422** | **0.005** |
| *lpl* | 1.00±0.14^a^ | 0.92±0.09^a^ | 0.48±0.09^b^ | 0.65±0.11^ab^ | 0.94±0.03^a^ | **cubic** | **y = 0.0295x^3^ - 0.1622x^2^ + 0.0405x + 1.1249** | **0.483** | **0.005** |
| *apoe1* | 1.00±0.01^bc^ | 0.78±0.03^c^ | 1.84±0.17^a^ | 1.27±0.06^b^ | 1.22±0.06^b^ | **cubic** | **y = -0.0719x^3^ + 0.54x^2^ - 0.9867x + 1.4232** | **0.293** | **0.059** |
| *apoa4* | 1.00±0.03^c^ | 1.02±0.10^c^ | 3.18±0.41^a^ | 2.50±0.33^ab^ | 2.20±0.13^b^ | **cubic** | **y = -0.1787x3 + 1.3744x2 - 2.415x + 2.1177** | **0.617** | **≤ 0.001** |
| *mttp* | 1.00±0.15 | 0.95±0.11 | 1.43±0.27 | 1.48±0.12 | 1.14±0.29 | cubic | y = -0.0767x^3^ + 0.617x^2^ - 1.2869x + 1.7246 | 0.180 | 0.184 |
| *fabp10a* | 1.00±0.11 | 0.49±0.06 | 1.46±0.21 | 0.61±0.17 | 1.33±0.12 | cubic | y = 0.0075x^3^ - 0.0233x^2^ - 0.0118x + 0.9319 | 0.085 | 0.691 |
| Lipid metabolism-related transcriptional factors | | | | | | | | | |
| *srebf1* | 1.00±0.05^a^ | 1.11±0.10^a^ | 0.43±0.04^b^ | 0.57±0.04^b^ | 0.62±0.05^b^ | **cubic** | **y = 0.0627x^3^ - 0.517x^2^ + 1.0623x + 0.4272** | **0.628** | **0.001** |
| *pparα1* | 1.00±0.11^a^ | 0.69±0.04^b^ | 0.41±0.03^c^ | 0.41±0.03^c^ | 0.42±0.07^c^ | **cubic** | **y = 4E-05x^3^ + 0.0668x^2^ - 0.548x + 1.4935** | **0.762** | **≤ 0.001** |
| *pparα2* | 1.00±0.05^a^ | 0.60±0.05^b^ | 0.18±0.02^c^ | 0.18±0.06^c^ | 0.12±0.02^c^ | **cubic** | **y = 0.0048x^3^ + 0.0526x^2^ - 0.6596x + 1.6254** | **0.747** | **≤ 0.001** |
| *pparβ* | 1.00±0.10 | 1.03±0.32 | 0.67±0.26 | 0.71±0.31 | 1.45±0.22 | cubic | y = 0.0918x^3^ - 0.6948x^2^ + 1.4333x + 0.1864 | 0.196 | 0.163 |
| *pparγ* | 1.00±0.07^b^ | 1.40±0.14^ab^ | 1.66±0.10^a^ | 1.39±0.11^ab^ | 1.24±0.13^ab^ | **cubic** | **y = 0.0293x^3^ - 0.3763x^2^ + 1.4099x - 0.0808** | **0.492** | **0.015** |
| *fxr* | 1.00±0.13^cd^ | 0.68±0.09^d^ | 2.78±0.28^a^ | 1.86±0.05^b^ | 1.62±0.12^bc^ | **cubic** | **y = -0.1704x^3^ + 1.3385x^2^ - 2.5688x + 2.197** | **0.512** | **0.003** |
| *lrh-1* | 1.00±0.06^a^ | 0.67±0.10^b^ | 0.45±0.06^bc^ | 0.33±0.04^c^ | 0.57±0.1^bc^ | **cubic** | **y = 0.0218x^3^ - 0.1083x^2^ - 0.1335x + 1.2137** | **0.618** | **≤ 0.001** |
| *hnf4α* | 1.00±0.16^a^ | 1.12±0.21^a^ | 0.28±0.04^b^ | 0.24±0.03^b^ | 0.28±0.04^b^ | **cubic** | **y = 0.0835x^3^ - 0.7001x^2^ + 1.4234x + 0.2627** | **0.642** | **≤ 0.001** |
| *lxra* | 1.00±0.11^b^ | 2.18±0.42^ab^ | 3.16±0.15^a^ | 3.22±0.19^a^ | 3.07±0.32^a^ | **cubic** | **y = 0.0023x^3^ - 0.2808x^2^ + 2.1348x - 0.8941** | **0.612** | **≤ 0.001** |
| Cholesterol biosynthesis | | | | | | | | | |
| *hmgcr* | 1.00±0.15^b^ | 0.34±0.06^b^ | 0.50±0.21^b^ | 1.02±0.36^b^ | 2.81±0.71^a^ | **cubic** | **y = 0.0415x^3^ - 0.0127x^2^ - 0.741x + 1.6265** | **0.636** | **≤ 0.001** |
| *dhcr24* | 1.00±0.13 | 0.83±0.10 | 0.66±0.10 | 0.83±0.12 | 1.09±0.13 | cubic | y = 0.0079x^3^ + 0.0129x^2^ - 0.3034x + 1.2955 | 0.283 | 0.090 |
| *lss* | 1.00±0.11^ab^ | 0.45±0.11^b^ | 0.67±0.24^ab^ | 1.05±0.25^ab^ | 1.32±0.10^a^ | **cubic** | **y = -0.0711x^3^ + 0.76x^2^ - 2.2654x + 2.5236** | **0.442** | **0.006** |
| *dhcr7* | 1.00±0.08^b^ | 1.04±0.09^b^ | 4.29±0.74^a^ | 5.32±0.44^a^ | 5.11±0.57^a^ | **cubic** | **y = -0.372x^3^ + 3.1537x^2^ - 6.3626x + 4.4917** | **0.846** | **≤ 0.001** |
| *sc5d* | 1.00±0.10^b^ | 1.03±0.15^b^ | 3.26±0.41^a^ | 3.70±0.40^a^ | 3.56±0.55^a^ | **cubic** | **y = -0.231x^3^ + 1.9271x^2^ - 3.7619x + 2.992** | **0.660** | **≤ 0.001** |
| Cholesterol transport-related | | | | | | | | | |
| *acat1* | 1.00±0.12^a^ | 0.93±0.13^a^ | 0.46±0.04^b^ | 0.36±0.05^b^ | 0.44±0.04^b^ | **cubic** | **y = 0.0492x^3^ - 0.3927x^2^ + 0.6911x + 0.6726** | **0.661** | **≤ 0.001** |
| *acat2* | 1.00±0.07 | 0.88±0.11 | 0.66±0.10 | 0.68±0.07 | 0.82±0.13 | cubic | y = 0.0176x^3^ - 0.1033x^2^ + 0.0304x + 1.0611 | 0.229 | 0.085 |

Data in a same row not sharing a same superscript letter are significantly different (*P*˂0.05). The significant regressions were marked in bold.

Supplementary Figure S1 The fatty oil composition of the oil sources used in this experiment (% TFA, mean).


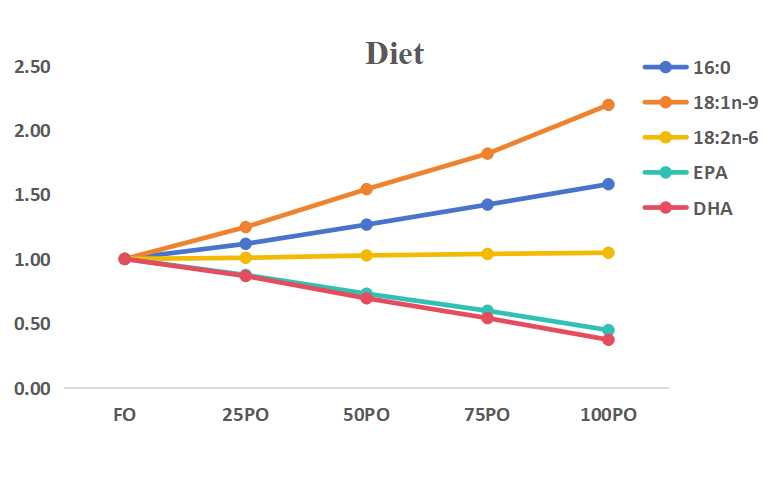

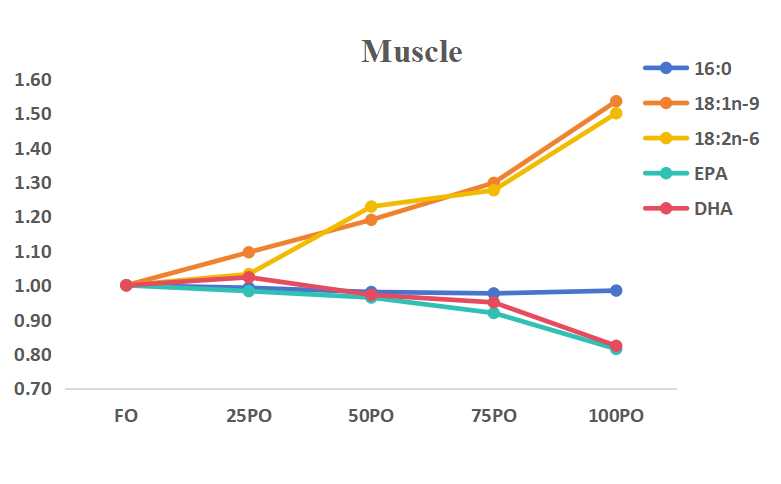


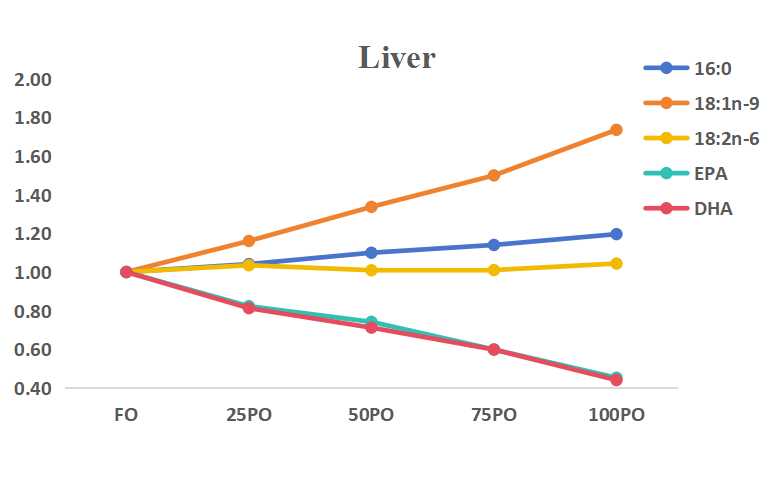

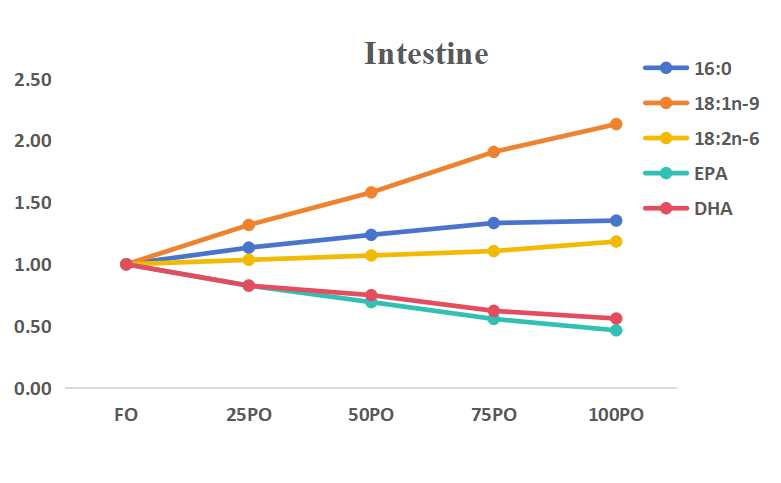


Supplementary Figure S2 Relative abundance of main fatty acids in experimental diets and the tissues of tiger puffer. The fatty acid contents in group FO were normalized to be 1.
